# Identifying animal taxa used to manufacture bone tools during the Middle Stone Age at Sibudu, South Africa: Results of a CT-rendered histological analysis

**DOI:** 10.1371/journal.pone.0208319

**Published:** 2018-11-29

**Authors:** Justin Bradfield

**Affiliations:** 1 Centre for Anthropological Research, University of Johannesburg, Johannesburg, South Africa; 2 Evolutionary Studies Institute and School of Geography, Archaeology and Environmental Studies, University of the Witwatersrand, Johannesburg, South Africa; Seoul National University College of Medicine, REPUBLIC OF KOREA

## Abstract

This paper presents the histological characterisation of a selection of worked bone artefacts from Middle Stone Age layers at Sibudu cave, South Africa. Histographic rendering is achieved using high-resolution Computed Tomography, which is non-destructive and facilitates three-dimensional histologic analysis. Excellent congruency in image quality was achieved with previous studies using this method. The results show that most of the artefact fragments contain mostly primary lamellar tissue, which is the bone tissue best adapted to withstand impact stresses. This indicates that bone with greater elastic properties was chosen. Histological characterisation allows the identification of animal taxa. Based on the sample analysed in this paper, Perissodactyla bone was used predominantly in the older layers at the site. Artiodactyla are represented throughout but appear far more frequently in the later (post-Howiesons Poort onwards) layers. Some of the Artiodactyla specimens have high proportions of Haversian tissue, reducing elasticity. The higher percentages of Haversian tissue in the post-Howiesons Poort artefacts relative to Holocene examples from southern Africa suggests that people may have started experimenting with bone from different animal taxa at this time and had not yet learned to eliminate the mechanically weaker secondary tissue. Apart from mechanical considerations, possible cultural constraints governing raw material selection is also explored.

## Introduction

Animal bones have been modified to make tools for a little over two million years [1–3]. Nevertheless, bone tools this old are extremely rare, becoming well-represented only in the Holocene. Between these two periods there is a brief florescence of bone tool manufacture at several Middle Stone Age sites in southern Africa, one of which, Sibudu, has yielded evidence for a variety of specialised implements [4]. Studies of these tools and other bone implements have focused on identifying the manufacturing processes and possible functions [4,5], with comparatively little attention paid to ascertaining the type of bone tissue and animal taxa represented by the bone implements, beyond the general size class of animal [5,6]. This is because most bone tools recovered from archaeological excavations are so pervasively modified that it is impossible to identify the type of animal from which they were made based on standard skeletal morphological markers. We can therefore only assume that the animal species targeted to fashion bone tools reflect the same species represented in the fauna record of food consumption.

In the absence of skeletal morphological markers modified bone may be identified using three techniques: 1) ancient DNA studies, which are accurate but expensive, time consuming and destructive [7]; 2) collagen isotope analysis, slightly less destructive and occasionally less accurate [8]; and 3) histological analysis, the least precise of the three but also least costly and destructive [9]. Bone histology has been used for many years to distinguish human from animal bone fragments. This is possible because human and animal bones are adapted to different mechanical requirements and therefore have different tissue structures and organisation [10–13]. Differential mechanical loading produces different responses in bone tissue formation such as secondary remodelling, osteonal banding and different tissue and vascular arrangements [12,14–17]. Just as the bones between humans and animals display adaptational differences, so too does bone of different animal taxa [18,19]. Recently this technique has been applied successfully to identifying the animal taxa represented in fragmented archaeological bones from several sites [9,20]. Although most histological studies thus far have used thin section micrographs, there has been a growing awareness of the benefits of high-resolution computed tomography (micro-CT) imaging over traditional thin section slides [21,22], not least because of the non-destructive nature of micro-CT.

In southern Africa, and indeed in many other parts of the world, there is ample historical and ethnographic evidence to indicate that among 19^th^ and 20^th^ century hunter-gatherers certain animals were favoured for tool manufacture over others that were readily available [23–28]. Such preferential selection was usually the result of deep-seated ideological connotations that people associated with certain animals [29–32], although this is not to discount other possible factors such as mechanical suitability. The extent to which bone tool manufacture in the South African Middle Stone Age was or was not framed within similar social constructs has never been explored. Neither has the question of whether people were selecting specific bone elements or portions thereof based on suitability to purpose. Establishing what bone tissue types and animal taxa are represented in the bone implement category at Sibudu is the logical first step in ascertaining whether raw material was selected expediently from what was brought in as food, or whether certain animal types were preferred for tool manufacture, and if so, whether this preference reflects the bone’s mechanical suitability for a desired task, or an intangible, social value.

In this paper I assess whether people at Sibudu during the Middle Stone Age were preferentially selecting animals from which to make tools or whether the bones from consumed prey were being used indiscriminately. Preferential selection is assessed in terms of mechanical suitability of the bone tissue to perform the hypothesized tasks attributed to these tools by previous studies [4,33]. I present the first micro-CT-rendered histological characterisation of completely worked bone tools from the ~65 to ~58 ka levels from Sibudu, South Africa. The material is attributed to the Howiesons Poort and post-Howiesons Poort technocomplexes at this site, although I also include one younger and one older specimen specifically to help mitigate the interpretative limitations arising from the small sample size of my study. I provide a qualitative and quantitative histological description and probable taxonomic identification of 20 bone tool fragments.

## Sibudu background

**Fig 1:**
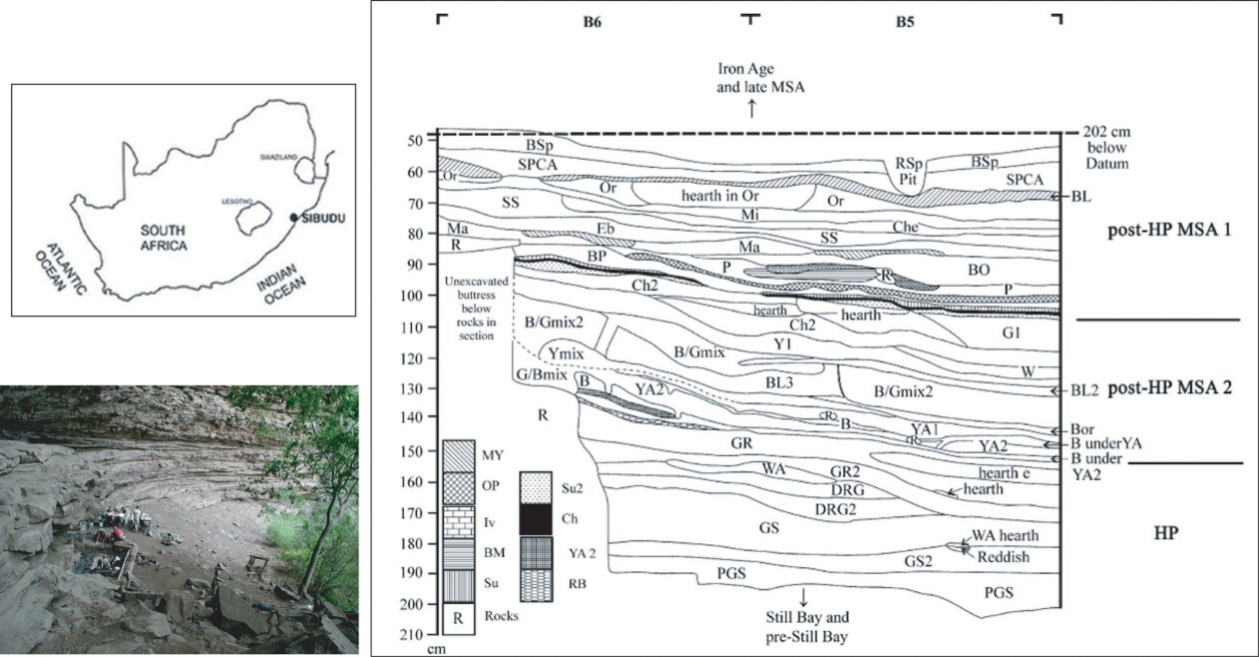
Map showing the location of Sibudu, the floor of the site and the relevant stratigraphy profile covering the Howiesons Poort and post-Howiesons Poort. Adapted from [41]).

Sibudu is one of the best preserved and best known Middle Stone Age sites in Africa. The site bears abundant evidence for the early manifestation of a suite of behaviours previously thought to be the domain of the Later Stone Age/Upper Palaeolithic. In this paper I concentrate on the faunal evidence from the Howiesons Poort and post-Howiesons Poort levels (Fig 1). Several changes in technology and subsistence strategies between the Howiesons Poort and post-Howiesons Poort have been described. Lithic technology changes from formalised reduction strategies in the Howiesons Poort to more expedient strategies in the post-Howiesons Poort, where different reduction strategies seem to appear and disappear in pulses [34]. In the post-Howiesons Poort there is evidence that bedding construction and other site maintenance activities intensified, suggesting that during this period the site was being occupied for longer periods at a time [35].

At the beginning of the Howiesons Poort the faunal assemblage is dominated by small bovids, particularly duiker, and a diverse range of other animal taxa preferring a closed, forested environment [6]. Humans were the primary contributor of macro-mammals to the site [6]. A bone point and small stone segments provide compelling evidence for bow-and-arrow hunting during this period [33, 36–38]. To be effective in thickets arrows would have had to have been much heavier than extant southern African historical examples [39]. Despite the evidence for arrows, the diverse range of small mammals, including carnivores and dangerous bushpig, have led some to postulate that they may have been caught using snares and traps [40]. Throughout the Howiesons Poort there is a steady decline in representation of primates, carnivores and animals preferring forested habitats with a concomitant increase in medium- to large-sized bovids [6]. By the latter half of the post-Howiesons Poort period the focus is clearly on larger ungulates preferring open grassland habitats [41]. Table 1 shows the minimum number of individuals (MNI) identified at Sibudu from the Howiesons Poort and post-Howiesons Poort. The data are extracted from Clark & Plug [41] and Clark [6] and, for the purposes of this paper, are presented according to taxonomic order and family. The shifting environmental conditions from closed forest to open grassland are confirmed by botanical evidence from the site [42,43]. All evidence points to increasing sedentism and reduced mobility during the post-Howiesons Poort period, related to environmental change and social impetus [34].

**Table 1:** List of taxa identified at Sibudu with cortical bone potentially suitable for tool manufacture. Data are adapted from [6] and [41]. HP is Howiesons Poort, p-HP is post-Howiesons Poort and MNI is minimum number of individuals.

Order	Family	HP MNI	p-HP MNI
Lagomorpha		11	3
Primate		15	1
Artiodactyla	Giraffidae	2	2
	Suidae	14	8
	Bovidae	74	28
Perissodactyla	Equidae	3	7
	Rhinocerotidae	3	0
Carnivora	Canidae	3	0
	Felidae	10	0
	Carnivora	7	0

Concomitant with the increasing occupation intensity over time at Sibudu there is an increasing number of burning events. The many hearth remains at Sibudu are interpreted variously as resulting from wood burning fires made to cook food, heat-treat lithics, and for site maintenance, which involved periodic burning of sedge bedding to kill parasites [44, 45]. Subsurface temperatures would have ranged from 177ᵒC to 240ᵒC, while surface temperatures may have reached 730ᵒC [46]. Burnt bone numbers increase moderately between the Howiesons Poort and the early phase of the post-Howiesons Poort, after which there is a dramatic increase in frequency in the latter phase of the post-Howiesons Poort [47]. The burnt bone is the result of human agency [48], occurring either as a result of waste disposal in hearths or incidentally through the frequent burning of bedding [47]. Other modifications to bone take the form of flaked, notched and grooved objects [49]. Part of this assemblage, mostly from the Howiesons Poort and post-Howiesons Poort but with two older examples from the Pre-Still Bay (>72 ka), was analysed and found to contain a variety of specialised pieces, including *pieces esquillèes*, wedges, smoothers and pressure flakers, previously unknown from Middle Stone Age sites [4]. Of the 23 pieces analysed by d’Errico and colleagues, 19 served utilitarian functions, while the remainder were notched with no apparent signs of use. The notched bones were fashioned from ribs, vertebrae and scapulae. The tools at Sibudu reflect a local tradition of bone tool manufacture as they are absent at contemporaneous sites where only the pointed bone tool variety has been recovered [4,5]. The presence of these tools at Sibudu cannot be explained by preservation factors, raw material availability, site function or environmental changes, but most probably result from peculiar innovations dependent on cultural transmission arising from demographic increase [4].

**Fig 2:**
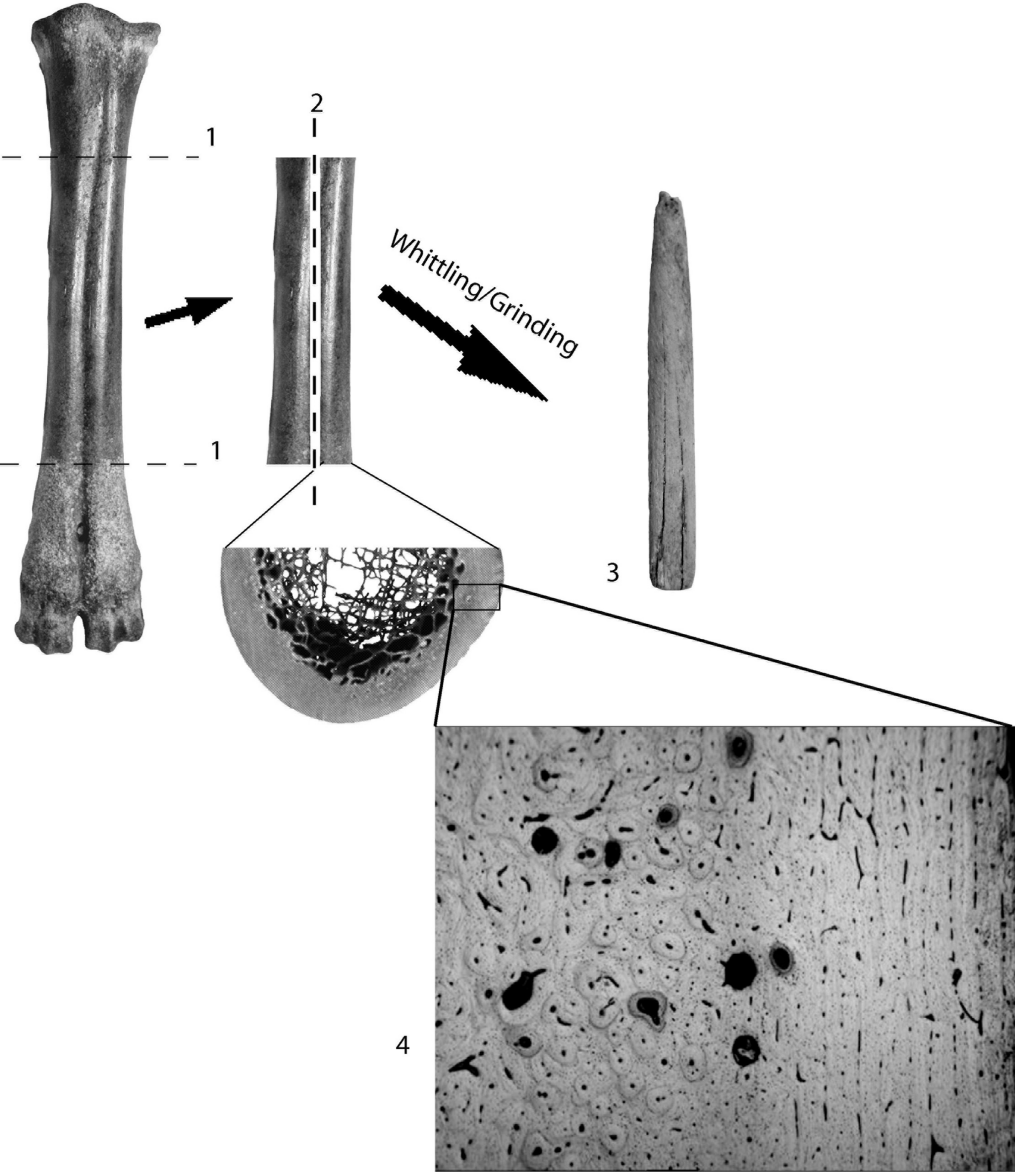
Bone tool manufacturing processes. 1) First the epiphyses are removed and then 2) the shaft is quartered by carving grooves down the length of the shaft and hammering a wedge into the groove. 3) Finally, the blank is whittled or ground into shape using a sharp lithic blade or abrasive stone surface. 4) The diameter of the end product will usually contain a representative portion of cortical bone, perhaps missing only the extreme endosteal and periosteal surfaces.

As far as the cylindrical tools go the bone manufacturing processes at Sibudu and other Middle Stone Age sites seem to follow the same initial chain of operations seen all over the world [50–52]. Fig 2 illustrates the typical manufacturing process for making cylindrical tools such as some of those from Sibudu. First, suitable long bones, such as metapodials, are chosen and the epiphyses are knocked off. Next, grooves are made down the length of the bone shaft and a wedge is used to split the bone apart. Finally, the suitable blanks are modified to their desired form either by grinding against an abrasive surface, whittled using a sharp lithic blade, or a combination of these two techniques [53]. The final product will contain a cross section of cortical bone, perhaps only missing the extreme endosteal and periosteal surfaces.

## Bone histology

Each vertebrate taxa has a unique combination of bone tissue characteristics, which develop in response to skeletal adaptations to mechanical activity usually governed by ecological or environmental factors [10,18]. Cortical bone may be either primary or secondary. Beyond this broad separation, bone histomorphology is usually characterised according to tissue structure and vascular arrangement [13]. Primary bone may present with several forms of tissue structure. The simplest of these, woven bone, is avascular and is found mainly in juveniles and at fracture sites [54]. Lamellar bone tissue on the other hand is found mainly in mature bone and consists of successive sheets of lamellae, which are layers of bone with parallel-orientated collagen fibrils [18,54]. When alternating layers of vascular lamellar and avascular non-lamellar bone occur together it is referred to as fibro-lamellar bone [13]. Primary lamellar bone may contain vascular canals housing blood vessels. These canals may present with a variety of arrangements, including radial, circular and reticular. Francillon-Vieillot et al. [10] provide some excellent graphical illustrations of these vascular arrangements. The most common vascular canal orientation is longitudinal. These, when surrounded by concentric rings of lamellae are called primary osteons [54]. When a vascular plexis containing canals variously orientated to form a brick-like formation occurs in fibro-lamellar bone it is referred to as plexiform and is a common tissue structure in many large mammals [54].

**Fig 3:**
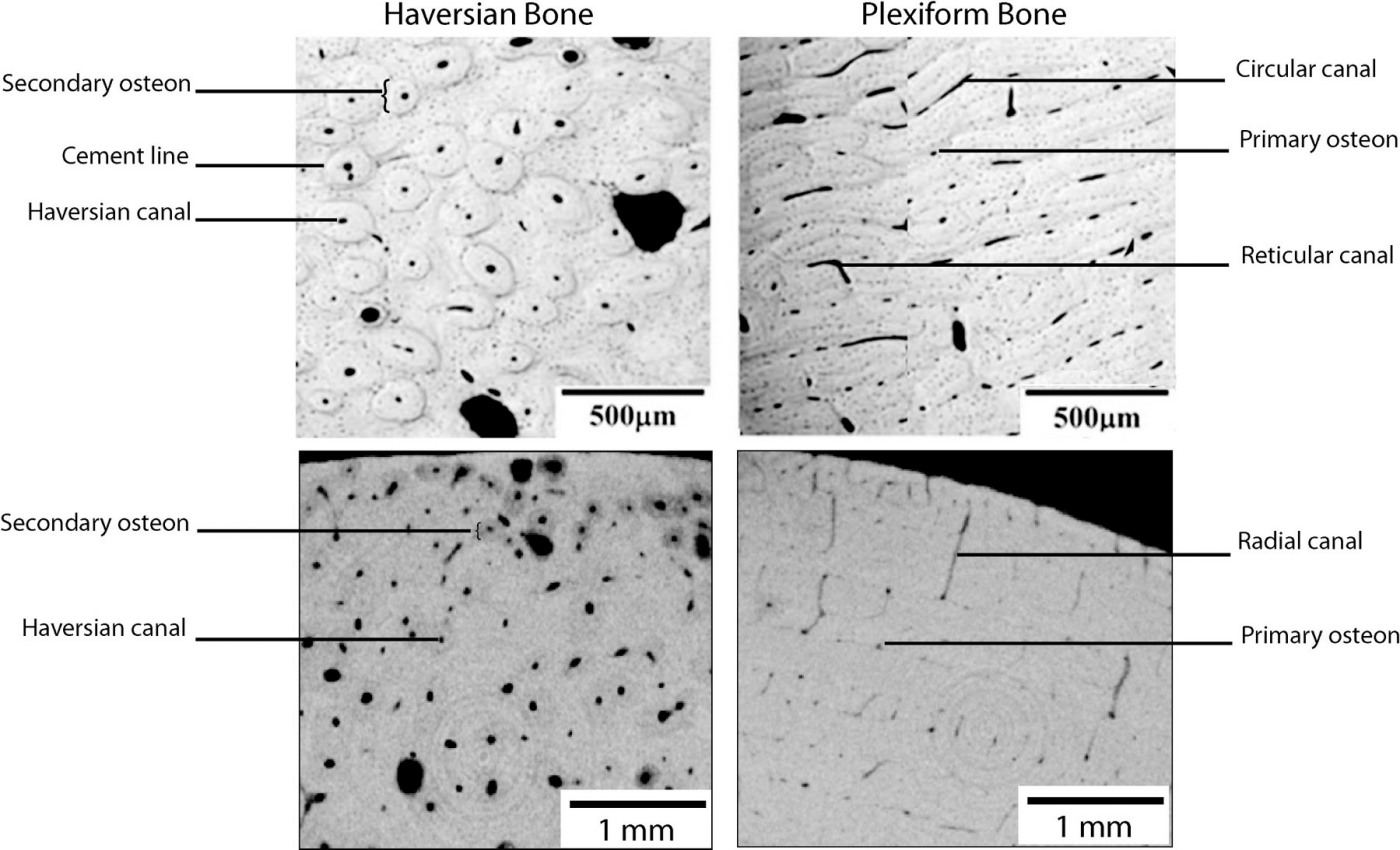
Comparison between primary and secondary bone tissue. The top row are typical histological thin section micrographs, while the images in the bottom row are derived from micro-CT And are taken from [22].

Secondary bone is less common than primary bone and forms as a result of remodelling processes occasioned by mechanical activity [54]. Secondary bone is characterised by larger vascular canals, surrounded by many more concentric layers of lamellae compared to primary bone [54]. The outer lamella typically forms a dense concentric line called a cement line, breaking the flow of the lamellae sheets. The whole structure is called a secondary or Haversian osteon [9, 55–57]. Bone comprising secondary osteons is referred to as Haversian bone. Fig 3 shows the differences between primary plexiform bone tissue and secondary Haversian bone tissue, with images derived from traditional thin section microscopy and micro-CT. Most non-human mammalian cortical bone will contain a combination of primary and secondary bone, with a variety of organisations and vascularisations differing between taxa ([15,58,59]; see also Fig 2, panel 4).

The potential of cortical bone microstructure to identify animal taxa was first recognised by Enlow & Brown who showed that species within different taxonomic orders tended to have dissimilar cortical micro-structural arrangements [60,61]. Subsequent comparative histology has shown considerable variation in bone tissue resulting from known modelling and remodelling processes [18,62]. Until relatively recently bone histology was used primarily to differentiate human from non-human bone [13,15] and to determine phylogeny in palaeontological fossils [63], although there is now a growing application to archaeological studies. Most recently, histology has been applied with great success in the identification of archaeological plant taxa [64]. In faunal studies histology has provided good results in differentiating between bones from different types of animals [9,65,66] and identifying the species of origin of tiny bone fragments used to temper pottery [67].

**Fig 4:**
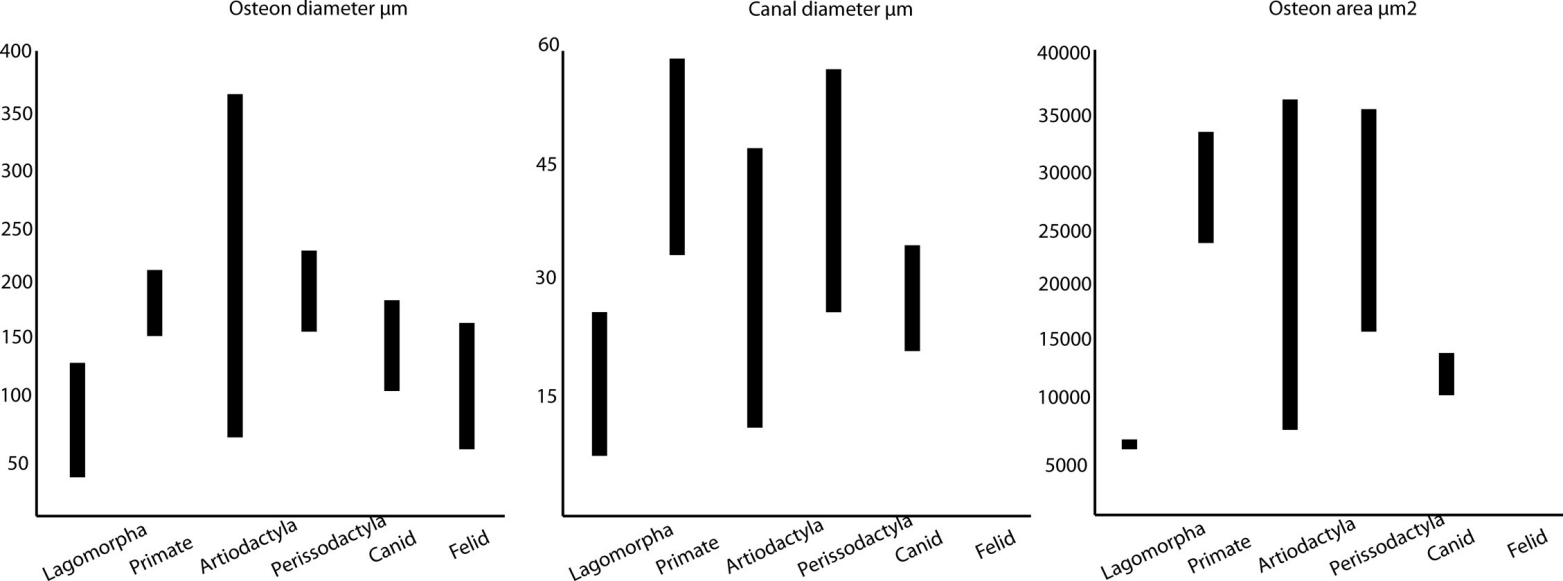
Graph showing how osteon dimensions differ between animal taxa.

Certain generalisations may be made regarding broad histologic differences between animals with different skeletal adaptations. For example, most small mammals tend to lack secondary osteons [60,68], while plexiform bone, osteon banding and radial canals are indicative of non-human, fast-growing mammals [13]. Despite some contradictory findings by different analysts in the type of bone tissue that occurs in different animal taxa [13,58], there is general agreement that both quantitative and qualitative assessments must be applied when analysing samples of unknown bone [13,15–17]. Qualitatively, bone will differ between taxa either in the type of tissue present or in the combination of tissues [16]. Secondary osteons appear to have the greatest quantitative metrics between taxa with canal diameter and osteon area being the most discriminating variables, although there is considerable overlap between taxa [16,17]. Table 2 presents the quantitative and qualitative characteristics of six mammalian taxa, while Fig 4 provides a graphical illustration of the different osteonal dimensions. Data are derived from Foot [69], Enlow & Brown [61], Singh et al. [58], Ricqlés et al. [70], Wang et al. [71], Martinaková et al. [16,17], Hillier & Bell [15] and Mulhern & Ubelaker [13]. Unfortunately, very few histological studies on non-human mammals have focused on southern African species. One notable exception described the femoral histography of eight South African mammal taxa, but only focused on the periosteal region of the cortical bone [72]. Nevertheless, these authors found excellent congruence between their specimens and existing descriptions in the literature.

**Table 2:** Quantitative and qualitative characteristics of six mammalian taxa. Data are derived from [13,15–17,58,61,69,70,71].

Taxon	Tissue structure	Vascular arrangement	Osteon density	Osteon diameter (μm)	Osteon area (μm^2^)	Canal diameter (μm)
Lagomorpha	Primary vascular tissue dominates with irregular large primary osteons in middle region. Periosteal lamellae alternate with vascular strata	Longitudinal. Large primary osteons may occur in middle region	Haversian-like primary osteons may occur in middle region	41–130	8339–8631	8–26
Primate	Primary circumferential lamellar grading to Haversian towards endosteal region	Primarily longitudinal with circular canals at periosteal border. Secondary osteons may occur densely in endosteal compact	Increased density from middle region to the endosteal border	139–215	23471–33000	33–59
Artiodactyla	Primary lamellar bone in periosteal region with Haversian bone towards endosteal region. Non-lamellar tissue may occur in endosteal region in **suidae**. Larger families like **giraffe** present with dense Haversian tissue in inner and middle compacta with avascular lamellae in periosteal region	Periosteal region has a well-organised plexiform arrangement with few scattered secondary osteons in old individuals. Secondary osteons densely clustered in middle and endosteal region, except in **suidae** which are non vascular in endosteal region and have greater number of resorption spaces	Density differs between families within this taxon	65–360	7410–36067	15–40 (Suid) 17–48 (bovid) 11–71 (cervid)
Perissodactyla	Primary fibrolamellar structure dominates. Haversian system may occur in endosteal region	Longitudinal and reticular grading to plexiform. Secondary osteons may be isolated or dense, arranged concentrically in the endosteal region. Density of primary osteons is usually greater than in Artiodactyla		158–238	15900–35506	26–58
Rodentia	Periosteal region is avascular while inner third of compacta contains circumferential lamellae. No Haversian system present	Basic pattern is reticular with radially arranged canals in inner third of compacta	n/a	n/a	n/a	n/a
Canidae	Primary vascular parallel tissue in periosteal region with Haversian system present in endosteal region. May have bands of avascular tissue	Periosteal region may have primary plexiform arrangement or radially arranged longitudinal canals	Secondary osteons may be isolated in middle region or be scattered here and increase in density towards endosteal region	117–183	10300–14034	21–34
Felidae	Circumferential lamellae in the narrow periosteal region with dense Haversian tissue interspersed throughout middle and endosteal region. May have bands of avascular tissue	Vascular canals are circular or bundled. Secondary osteons are smaller than canidae. Primary and secondary canals may occur together in same compacta		65–163	No info	No info

Histological structures of taxonomic value are visible at 30X-100X magnification [9], although typically 100X magnification is preferred [72]. Different methods have been used to acquire histographs. Transmitted light microscopy is the most common, particularly in the biological sciences. Another method, micro-radiography, provides excellent images and is able to clearly identify Haversian osteons which appear as darker patches around canals due to their lower degree of mineralisation [57; 73,74]. Micro-radiography typically provides a 1**μ**m resolution at 30X magnification making it just as suitable as transmission light microscopy [75]. The main limitation with these two methods is that they provide only two-dimensional images, making recognition of certain histological features difficult [76].

To overcome this limitation micro-CT is being increasingly applied to histological analyses of bone and other tissues [11, 21,63,77–85]. Most of these studies have found micro-CT to be superior to traditional histological techniques, at least as far as quantitative analyses are concerned [80,86]. One of the great advantages of micro-CT over traditional thin sectioning is that it allows a larger area to be viewed, which in turn allows an analyst to characterise not just one region but the entire cortical matrix. There is, however, a necessary trade-off between higher resolution and greater field of view [80]. The resolution provided by micro-CT is not as high as other methods, being in the range of 5 **μ**m. This means that certain structures such as cement lines, individual lamellae etc. are not visible (see [12], [54] for metrics of these features). However, features such as lacunae, primary and secondary osteons etc. are clearly visible [80,86]. Synchrotron radiation allows CT images to achieve a sub-micron resolution, but the reduced field of view negates the analytical advantage for taxon identification, as it would require the digital stitching together of multiple scanned volumes [87].

## Factors affecting bone histological identification

Apart from the challenges faced by image acquisition, the ability to accurately identify animal taxa based on bone histology is hampered by other issues that must be taken into account. Bone histomorphology may be affected by a number of factors including the age of the individual, the skeletal element sampled, pathological conditions that afflicted the animal during life, and post-depositional alterations arising from heat, microbial action, and other factors responsible for bone demineralisation. Any histological study of worked or fragmented archaeological bone must make certain fundamental assumptions before it can proceed. First, we must know whether the bone derives from an adult or juvenile individual as the tissue structure and organisation can differ dramatically. Based on the faunal data from Sibudu we know that people in the Howiesons Poort and post-Howiesons Poort were preferentially targeting adults for food consumption [6,41], a finding that agrees with the predictions of optimal foraging theory [88]. Although juveniles are present in the fauna remains, they do not exceed 10% of the sample at any one time [6,41].

Secondly, we must know what skeletal element and what part of the skeletal element the bone tool was made from, as the microstructure may differ dramatically depending on where in the animal’s body a bone derives [18]. When working with fragments of completely modified bone it is usually impossible to directly verify the exact skeletal element. At best we can usually only tell if it is a long bone, a flat bone or an irregular bone. Even if we can recognise a long bone it is possible that the microstructure will differ between the anterior and posterior portions of the shaft depending on the particular mechanical load experienced during the life of the animal [89,90]. Based on analogy to a South African Later Stone Age bone tool fabrication site [50] and Mesolithic bone tool manufacturing reconstructions from elsewhere in the world [52], however, we may suppose that metapodial diaphyses of medium to large game would have been the preferred skeletal element to use for bone tool production at Sibudu. These are the easiest skeletal elements to modify into pointed cylindrical implements. Most of the Sibudu bone tools examined here are cylindrical shafts. Unlike most other long bones the bone tissue structure and vascular arrangement of metapodials does not differ significantly within an individual bone or between metacarpals and metatarsals [91].

Thirdly, normal bone pathology can be assumed based on the relative scarcity of wild animals evincing abnormal pathology [92]. Some pathological conditions can be identified from micro-CT images [93] so need not be a hindrance to histological analyses. Likewise, mineralisation and microbial action are easily identified on computed tomographs, showing up as bright spots or irregular tunnels of high density [94]. It is expected that all archaeological bone will have experienced some degree of microbial or other taphonomic alterations, but these are dealt with in depth in the literature and are therefore easily recognisable [9,94,95]. One factor that is particularly relevant to the Sibudu bones is heat alteration. As already mentioned above, Sibudu experienced frequent burning events, which have affected many of the faunal remains [47,48]. Burning, and contact with associated calcitic ash, may alter bone microstructure by causing cracking, shrinking and slight tissue deformation [14,22,96,97,98]. Recognition of histologic structures, however, is still achievable in bone burnt at less than 600°C [14,74,97]. Considering that the average camp fire using South African wood taxa seldom exceeds 500°C we should not expect heating events to adversely affect bone histology at Sibudu [46,99]. This is confirmed by the study by Hanson and Cain [45] who presented histological thin sections from Sibudu with bone microstructure clearly visible.

The final factor affecting histological analysis is the degree of alteration of the cortical bone during tool manufacture. The endosteal region of the long bones of some animals contains different tissues and organisation from that which is present in the periosteal region (see Fig 2). If cortical bone is reduced to the extent that an entire region is removed, then it may not be possible to identify the animal accurately [15]. For this reason, Enlow [18] conceded that histology can more profitably be used to rule out certain taxa rather than to positively identify the animal taxa of an unknown piece of bone. With respect to the cylindrical shaft fragments at Sibudu we must assume that people would have undertaken to reduce the bone only as far as was necessary to perform the desired function. When we consider bone tool manufacture at other sites in South Africa during the Later and Middle Stone Age there is no reason to suppose that more complex reduction strategies were followed at Sibudu [5,50,100].

## Method

The worked bone artefacts from Sibudu were excavated by Lyn Wadley under Amafa permit #0007/09 and are formally accessioned at the KwaZulu-Natal Museum. The material included here is on long-term loan to the Evolutionary Studies Institute, University of the Witwatersrand. Permission to study the Sibudu material was granted by the permit holder and the repository curator. No permits were required for the described study as under the South African National Heritage Resources Act (Act 25 of 1999) these are only necessary when conducting destructive sampling or exporting samples for analysis. MicroCT is an entirely non-destructive and non-invasive technique, suitable for use of archaeological and fossil specimens.

**Fig 5:**
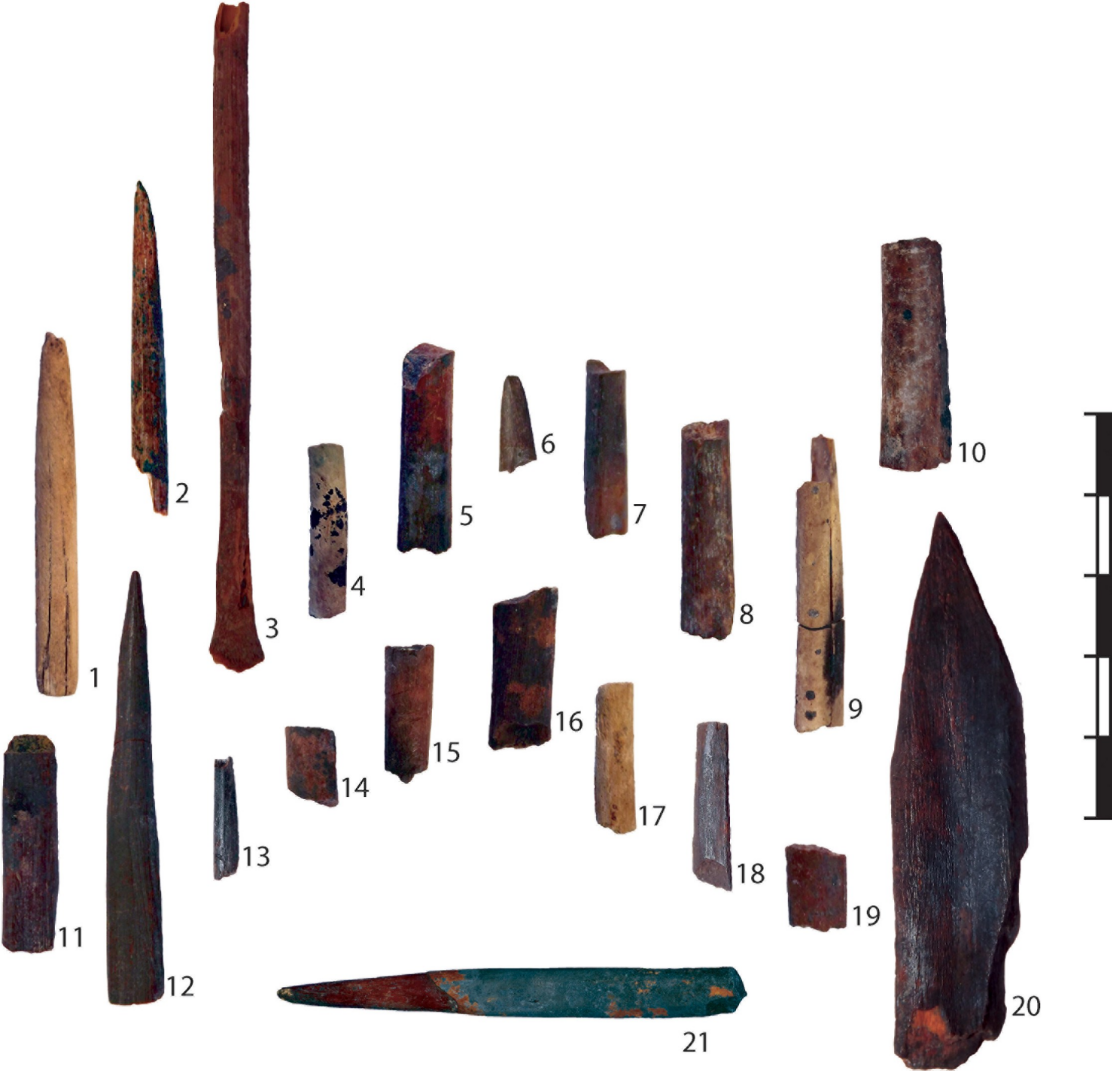
The sub-set of cylindrical bone tool shafts from Sibudu reported on in the paper.

The archaeological specimens were chosen based on two criteria: 1) the size of the specimen, and 2) the overall shape of the specimen. Artefacts that were too large, of which there are many examples among the worked bone assemblage at Sibudu, would not be able to be brought close enough to the x-ray emitter to achieve the necessary resolution and magnification to allow histological examination. As a result, my focus here is on a sub-set of the larger worked bone assemblage at Sibudu, and includes small cylindrical or elliptical shaft fragments morphologically akin to the Sibudu arrowhead and which could have served the same or similar purpose (Fig 5). None of the worked bone tools have been assigned formal accession numbers and so they are referred to here by their provenience number (see Table 3).

**Table 3:** Qualitative and quantitative histological results listing the probable animal taxon identification for each artefact. Osteon measurements are for secondary osteons only.

Sample #	Provenience	Techno-complex	Age (ka)	Supposed function	Taphonomy	Osteon diameter	Osteon Area	Histology description	Probably taxa
1	Surface	Iron Age	<2	Arrowhead	N/A	200–270	31400–57226	Periosteal and medial compacta comprise fibrolamellar plexiform bone. Endosteal compacta comprises primary longitudinal bone with scattered secondary osteons	Artiodactyla
2–3	C5b PGS	HP	64.7±1.9	Awl	Cracking & Digenetic Dissolution	260	56700	Primary vascular lamellar bone. Canals are orientated longitudinally and circumferentially. Isolated Haversian canals near the endosteal region. Anterior region contains avascular bone.	Primate
4	B5c WA	HP	-	-	Microbial	120–190	11304–20096	Primary vascular fibro-lamellar bone with radially arranged longitudinal canals and reticular and circular canals.	Perissodactyla
5	C5b GR1	HP	<61.7±1.5	-	Mineralisation, Heating & Microbial	/	/	Primary vascular fibro-lamellar bone with radial canals and longitudinal canals in a circular arrangement. In the middle and endosteal compacta the radial canals are replaced with reticular canals and bundled longitudinal canals.	Indeterminate
6	JS B2/3 BM	p-HP	<58.0 ±2.1	-	N/A	30–40	1200–1256	Dense primary vascularised fibro-lamellar bone. Longitudinal canals in bundled arrangement.	Indeterminate
7	C5b PGS	HP	64.7±1.9	-	Mineralisation	170–270	22686–57226	Periosteal compacta contains poorly vascularised primary lamellar bone with circumferentially-arranged longitudinal canals. The medial compacta is more highly vascularised consisting of primary lamellar bone with bundled longitudinal canals and reticular canals. Few isolated Haversian osteons occur between periosteal and medial compacta.	Indeterminate
8	B5b B under YA2	p-HP	<58.0 ±2.1	-	Digenetic Dissolution & Mineralisation	150–280	17662–61544	Thin periosteal region composed of plxiform tissue. Dense Haversian bone with fibro-lamellar structure in medial and endosteal compacta.	Artiodactyla
9	B4b YP	p-HP	<58.0 ±2.1	-	Microbial	170–200	22686–31400	Primary longitudinal canals in circular arrangements grading to banded arrangements away from the periosteal region. Reticular canals also present. Some isolated secondary osteons visible.	Carnivora/ Artiodatcyla
10	C4a BS14	pre-SB	-	-	Mineralisation & Cracking	120–210	11304–34618	Thin avascular periosteal region followed by thin circumferential band of Haversian osteons. Remaining compacta is fibrolamellar primary longitudinal bone in bundled arrangement with many reticular canals and scattered secondary osteons.	Perissodactyla
11	C4c PGS2	HP	>64.7±1.9	-	Mineralisation	/	/	Fibro-lamellar bone with plexiform arrangement in the periosteal region. The middle compacta is primary longitudinal bone with reticular canals.	Artiodactyla?
12	C4d PGS2	HP	>64.7±1.9	Awl	Mineralisation	/	/	Primary vascular fibro-lamellar bone with longitudinal canals in a radial arrangement. Reticular canals also present.	Indeterminate
13	B5c PGS	HP	64.7±1.9	Pin	Mineralisation	/	/	Sparsely vascularised, non-lamellar bone with some primary bundled longitudinal canals and scattered radial and reticular canals.	Indeterminate
14	B4b GS2	HP	63.8±2.5	-	Mineralisation & Microbial	/	/	Periosteal compacta has primary radial canals and circumferentially arranged longitudinal canals. Medial compacta is densely vascularised with reticular canals and bundled longitudinal canals.	Perissodactyla?
15	C6d GS	HP	61.7±1.5	-	Weathering	/	/	Periosteal region is non-lamellar and sparsely vascularised. The medial compacta is fibro-lamellar, heavily reticulated and contains circumferentially and radially arranged primary vascular canals. No secondary remodelling is visible.	Perissodactyla?
16	B5C B/GM2	p-HP	57.8±2.3	-	Cracking	/	/	Outer compact is lamellar with primary radial canals and circumferentially arranged longitudinal canals. Medial compacta is fibro-lamellar primary reticular with bundled longitudinal canals. Inner compacta appears to be avascular or sparsely vascularised lamellar bone.	Perissodactyla
17	C4d YA2	p-HP	<58.0 ±2.1	-	Digenetic Dissolution	/	/	Periosteal compacta comprise fibrolamellar plexiform bone. Medial compacta comprises large reticular and bundled longitudinal vascular spaces exaggerated through heating. Isolated Haversian osteons may be present.	Artiodactyla
18	C5c SPCA	p-HP	<58.0 ±2.1	-	Digenetic Dissolution & Mineralisation	220–270	37994–57226	Outer compacta with primary radial canals and some circumferentially arranged longitudinal canals. Inner campacta is densely Haversian fibro-lamellar bone with few scattered primary longitudinal osteons.	Carnivora/ artiodactyla
19	C5a GS	HP	61.7±1.5	-	Mineralisation	/	/	Primary reticular canals with longitudinal canals arranged circumferentially and radially. Not true plexiform system. No secondary tissue present.	Perisodactyla?
20	C5c GS2	HP	63.8±2.5	Awl	Digenetic Dissolution	/	/	Sparsely vascularised non-lamellar bone with primary longitudinal canals arranged circumferentially in outer compacta and bundled in medial compacta. Inner compacta comprises primary radial canals.	Indeterminate
21	B5d GS	HP	61.7±1.5	Arrowhead	Mineralisation	/	/	Primary longitudinal canals in circumferential and bundled arrangements. Some reticular canals present.	Indeterminate

Image data from the bone points were acquired using an X-Tek microfocus X-ray computed tomography (Nikon Metrology XTH 225/320 LC dual source industrial system) machine. All bone specimens, both archaeological and modern comparatives were scanned at 70 kV and 120 **μ**A with a 225 kV rotating target. Two thousand projections were acquired at one frame per second with a frame averaging rate of one. The scans achieved a 5 **μ**m resolution with an effective magnification of 30X. Each volume consisted of a 10 mm^3^ block incorporating the widest point of the bone fragment. Following recent successful protocols [38,101] specimens were scanned in air without the use of any contrasting agents. Comparative animal bone was sourced primarily from the modern fauna collection at the Wits Palaeosciences Centre, specimens BPI541, BPI911, BPI1120. The skeletal elements chosen from the comparative specimens were metapodials and femurs. In the case of very thick bone, like the giraffe, the bone was scanned in two acquisitions that were then digitally stitched together.

The transverse sections of each acquisition of Sibudu bone were analysed against both the modern comparative CT scans and published histological sections from the literature [10,72,91,102]. In particular, I relied on previous histology studies that used micro-radiography, BSEM or micro-CT for comparable images and resolutions [71,73,103,104]. There is much variation in descriptive nomenclature in the literature, so I follow the same descriptions as those used for other southern African animal taxa [72]. I rely on three-fold criteria of tissue structure, vascularisation, and metric data. Identification of taphonomic alterations relies on existing literature describing these phenomena [94,95, 105–107].

## Results

**Fig 6:**
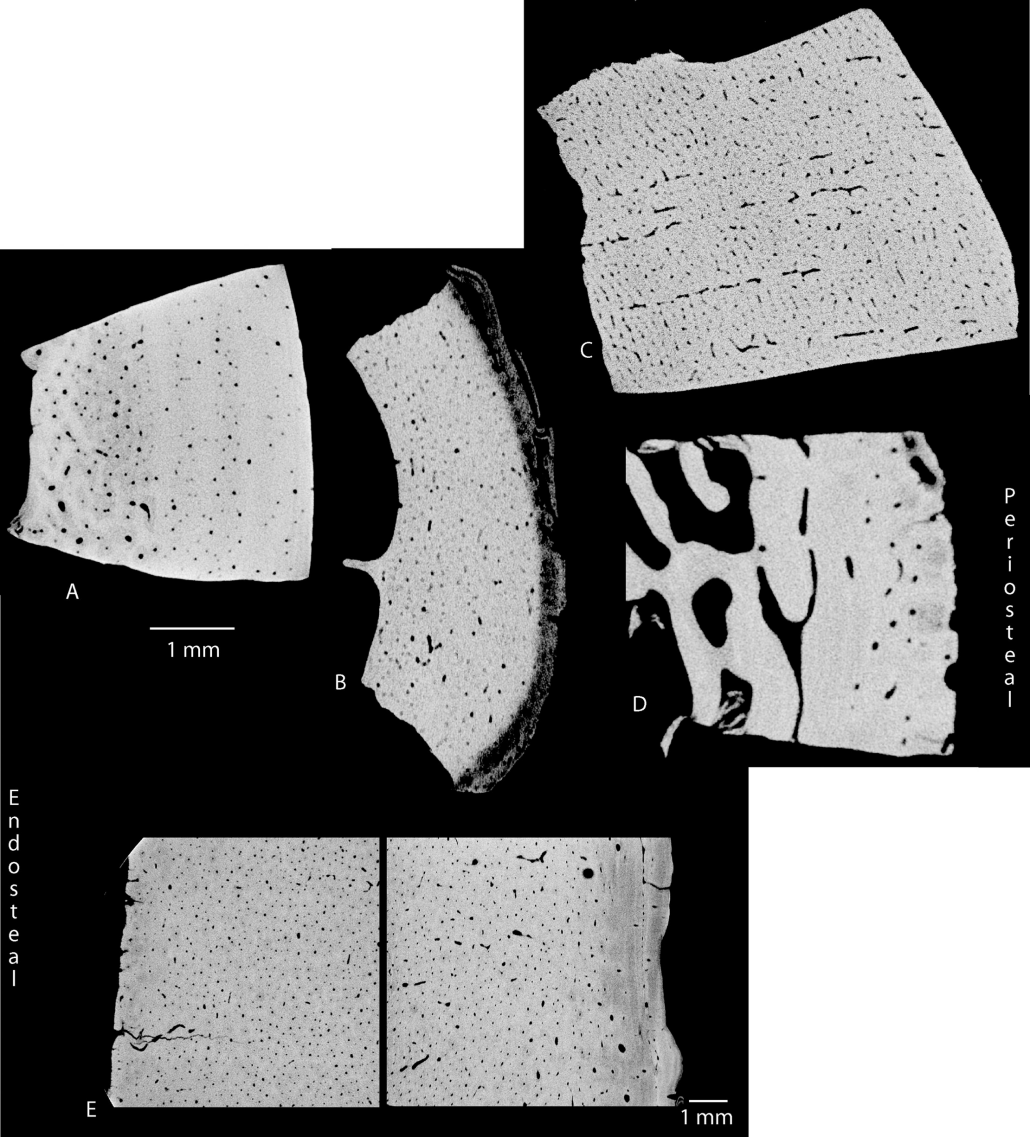
**Some comparative CT-rendered histologic slices through cortical humerus bone from: A) primate, B) canid, C) ostrich, D) felid, and E) giraffe.** Each image is orientated with the perisosteal surface on the right.

**Fig 7:**
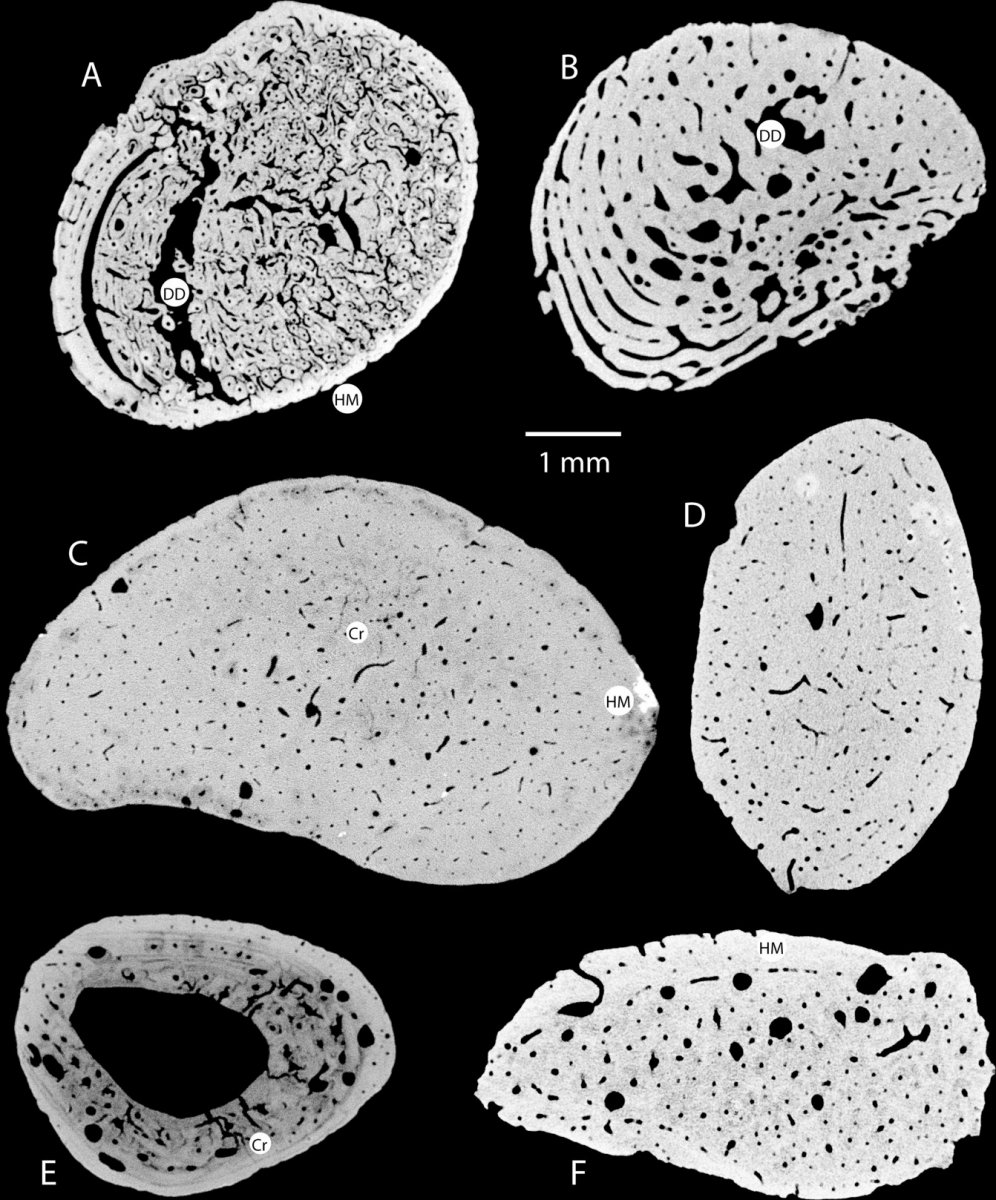
**Examples of CT-rendered bone histographies from Artiodactyla (A, #8 and B, #17), Perissodactyla (C, #10 and D, # 19), primate (E, # 2), and carnivore (F, # 18).** Cr indicates heat-induced cracking; DD indicates digenetic dissolution; and HM indicates hyper-mineralisation.

Excellent congruence in image quality and visualisation between the images obtained here and previously published micro-CT acquisitions was achieved. Fig 6 shows some of the comparative specimens that were scanned. Primary bone is clearly distinguishable from secondary bone, as are the different types of primary bone tissue and vascular patterns. A selection of micro-CT scans of the Sibudu bone implements is presented in Fig 7. Noticeable in these images is a degree of taphonomic alteration absent in the comparative specimens. Taphonomic alterations are evident in 18 specimens (Table 3). Digenetic dissolution and hyper-mineralisation, most likely associated with the proximity to a hearth and its resulting ash [98],occur most frequently. In all cases, taphonomic alterations were easily identifiable and did not significantly hamper histological analysis. Digenetic dissolution (DD) presents as exaggerated vascular spaces (Fig 7A & 7B), whereas hyper-mineralisation (HM)presents as bright areas in the tomographs (e.g. Fig 7C & 7D), These are due to the leaching of collagen out of the bone and soil minerals into the bone respectively. In almost all the scans it is possible to orientate the endosteal and perioseal sides of the bone based on the curvature of the internal tissue organisation; the larger part of the arc indicating the periosteal surface. The quantitative and qualitative results are presented in Table 3. Haversian tissue is identified by the presence of large resorption spaces and a distinctive, though sometimes faint, dark area surrounding a vascular canal, indicative of a Haversian osteon [74]. Occasionally, due to mineralisation processes, these have a white area. In rare examples individual lamellae separations are apparent (e.g. Fig 7E).

Secondary Haversian bone tissue was identified in nine of the 20 specimens (45%; Table 3). In 78% of these (n = 7) the proportion of Haversian tissue does not exceed 20% of the total surface area. Haversian osteons appear either isolated and scattered, or as concentric bands near the periosteal surface. A distinct plexiform configuration is evident in four specimens (Table 3), while the majority of specimens consist of primary lamellar bone tissue. Fig 7 presents selected micro-tomographs showing the different histology according to taxa. Plexiform arrangement is clearly visible in Figs 7A and 6B, while the darker Haversian osteons may be seen in Fig 7C, 7E and 7F. The micro-tomographs of each of the scanned Sibudu specimens, as well as each of the comparative specimens are provided in S1 File. Specimens that were not identifiable to taxa contained large amounts of primary bone.

Probable taxonomic identification was achieved in 13 (65%) specimens (Table 3). There is an equal representation of the orders Artiodactyla and Perissodactya, followed by carnivore and primate. I was unable to identify seven specimens due to unclear or ambiguous tissue structural organisation. Artiodactyls are the most prominent mammal taxa occurring at Sibudu, so it is not unexpected to find them well represented in the worked bone category. There are, however, many diverse species incorporated within this taxon, some of which may have slightly different histomorphometrics from that typically associated with this taxon, which is based on cattle and other large bovine bone. For example, giraffe femoral bone has a very different micro-structure (Fig 6), whereas suids differ from cattle only slightly. Two specimens presented here as artiodactyls could reasonably be attributed to suids (#9 and #11), although, in the case of #9, many features shared with the order carnivora are also present, rendering precise identification impractical. Suids are reasonably well represented in the Sibudu fauna (see Table 1) and could easily have been used to make tools. Two possible carnivore bones are represented and one primate bone. The putative carnivore bones appear to most closely resemble canid, but the histomorphology is not precise enough to exclude Artiodactyla bone, some genera of which have regions of similar structure. Of particular interest is the use of Perissodactyla to fashion bone implements. Although they occurred in equal number to artiodactyls, mammals of the Perissodactyla taxon occur far less frequently in the unmodified fauna at Sibudu (cf. Table 1). The Perissodactyla taxon also contains fewer species in southern Africa than Artiodactyla, with the most notable being zebra, and rhinoceros.

If we view the results by age we see that the Howiesons Poort and post-Howiesons Poort specimens display certain differences, despite the overall small sample size and disparity between Howiesons Poort and post-Howiesons Poort samples. Among the bone artefacts analysed here Perissodactyla occur exclusively in the older layers (a single example from a pre-Still Bay level and the remaining four from the Howiesons Poort levels). Artiodactyla and the two possible carnivore specimens occur in the post-Howiesons Poort levels, with only one example of an artiodactyl from the Howiesons Poort and one from the overlying Iron Age levels. Taken at face value it appears that there is a shift from Perissodactyla to Artiodactyla over time; but this is to ignore the nine indeterminate specimens, most of which occur in the Howiesons Poort. Some of these indeterminate specimens could be suid, but there are not enough diagnostic features to be certain. As mentioned above, the unmodified fauna from the Howiesons Poort shows a higher frequency of smaller animals, with primates and carnivore declining in frequency over time. The fact that no Perissodactyla bone was recognised among the post-Howiesons Poort bone tools may be simply a factor of small sample size rather than a conscious avoidance of this taxon. Related to species representation is the representation of bone tissue. The two specimens evincing high percentage of secondary osteons both come from the post-Howiesons Poort layers, but, apart from these two, the selection of bone tissue does not appear to change significantly over time.

**Fig 8:**
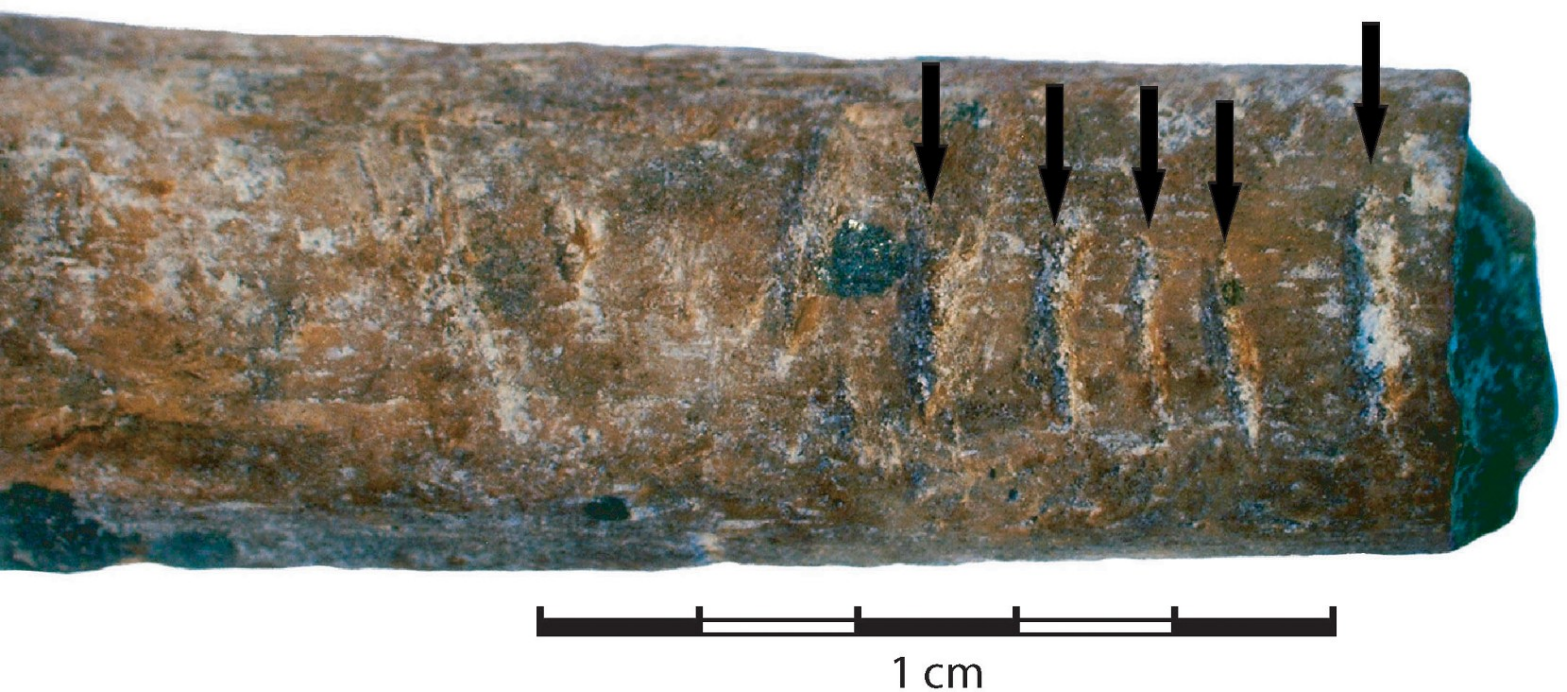
Notched piece from pre-Still Bay layers (C4a BS14).

**Fig 9:**
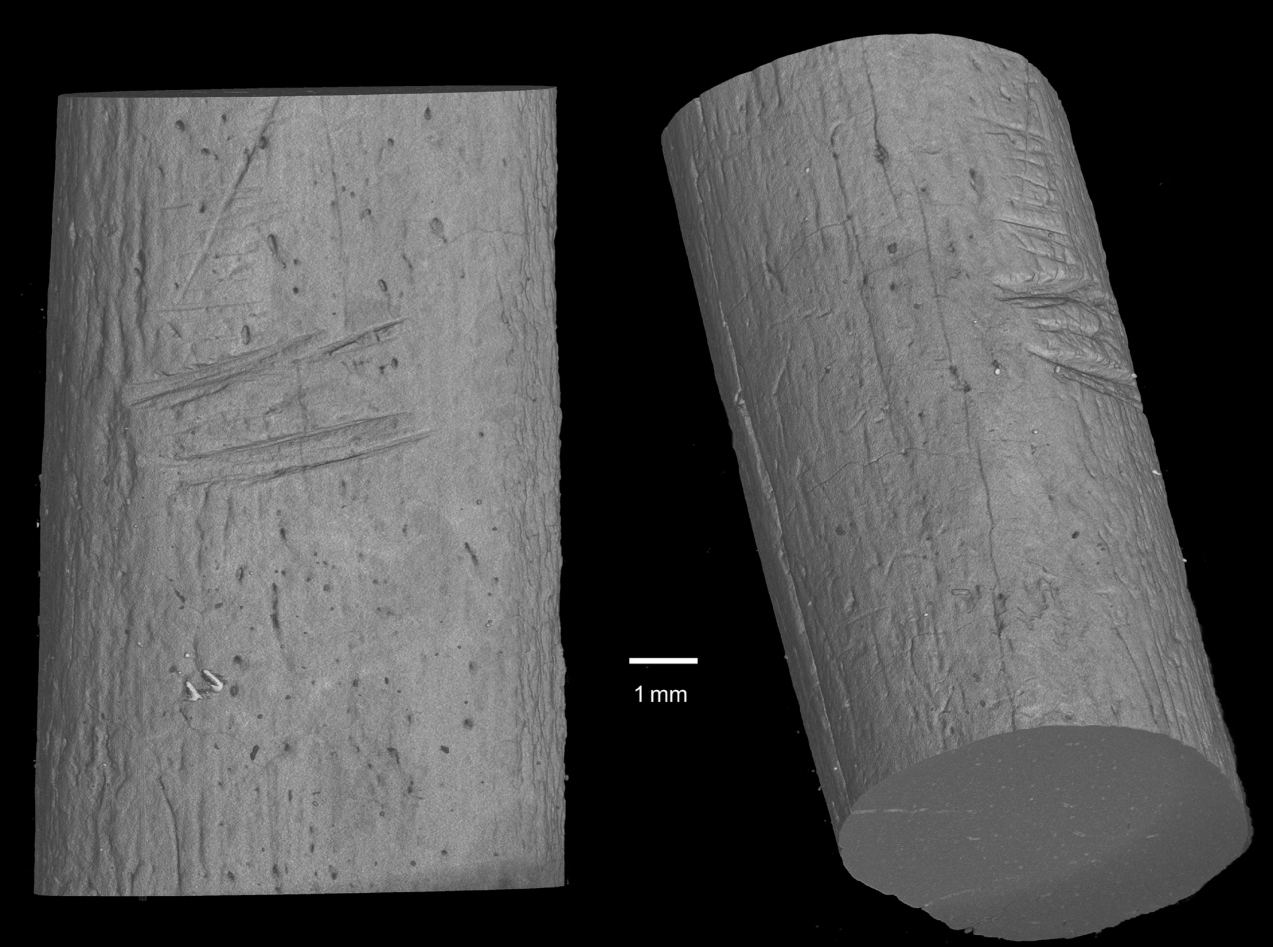
Three-dimensional tomography showing cut marks on shaft #15 (C6d GS). These cut marks are consistent with arrow retrieval marks identified elsewhere.

Incidental to the main objective of the microCT scanning was the identification of surface markings on two of the bone specimens (Figs 8 & 9). These markings appear as deep ‘v’-shaped incisions, probably produced with a sharp lithic edge. The specimen from the Pre-Still Bay layers (#10) has a series of five extant notches along one side of its shaft (Fig 8). It is probable that the complete implement from which this fragment derives had more notches. The second specimen (#15) has a series of concentrated cut marks on one side of the shaft (Fig 8). These cut marks overlie the manufacturing striations associated with fashioning the cylindrical shaft, appear less deliberate than on #10, but not entirely random as there are clearly two concentrations.

## Discussion

The twenty bone artefacts chosen for this study are merely a sub-set of the worked bone sample recovered from Sibudu Middle Stone Age layers. As such, any inferences from these results must be proffered with caution. Of the five tools included here that previously have been analysed for use-wear, all are considered to have performed a piercing function, either as an awl or an arrowhead. Of the remaining shaft fragments only two provide an indication of possible function, namely the notched and cut-marked bones Although one other notched piece has been described for pre-Still Bay levels at Sibudu, the notches on Fig 8 more closely resemble those described for much younger Howiesons Poort and post-Howiesons Poort layers [4]. The cut marks on Fig 9 likely represent ‘retrieval marks’ caused by cutting out an arrowhead lodged in a carcass [108–110]. It is possible then that this specimen represents part of a second bone arrowhead. Assuming then that most of the Sibudu shaft fragments would have served a piercing or perforating function and would have been subject to specific forms of stress, we can reasonably ask whether the bone chosen to make these tools was optimally suited to these functions.

The overall strength and fracture-arresting properties of cortical bone depends on its microstructure [84]. Different tissue structures and organisations develop in bone as a result of differing stresses experienced *in vivo*. For example, regions experiencing high compressive stresses will develop Haversian systems, whereas regions experiencing low stresses develop fibro-lamellar plexiform bone [90,104]. There have been many studies in the medical and biomechanical fields that have assessed the various breakage properties of cortical long bone diaphyses (see references in [111]. These studies have focused almost exclusively on the two most common types of bone tissue, namely primary plexiform bone, which commonly occurs in cattle and other large, fast-growing mammals, and secondary Haversian bone, which is the principal bone tissue found in humans. It has been found that, in general, owing to its unique structural mechanics, larger vascularity and orientation of collagen fibrils, the fatigue and tensile strength of Haversian bone is weaker and more prone to breakage than plexiform bone [55,56,112–118]. Haversian bone is adapted to compressive stresses and lacks the elasticity and fatigue strength of plexiform bone [89,90,116,119]. Pure lamellar bone in turn is mechanically superior to fibro-lamellar /plexiform tissue [13], although, in precisely what sense it is superior is not explained. There is a wide variety of primary bone vascular arrangements [10] each of which may be expected to influence the mechanical responses to stress and loading. Ascenzi and colleagues [120] tested compression and shearing responses in primary bone of three vascular arrangements, namely, longitudinal, radial and reticular arrangements. They found that bone with radially arranged canals has the greatest elasticity and resists both compression and shearing stresses better than the other two vascular arrangements. This is followed by reticular arrangement and longitudinal arrangement respectively [120]. Woven bone on the other hand, although far less is known about the mechanical properties of this tissue type, is believed to have the greatest stiffness properties, but be more brittle than other bone tissues [54].

Bone may experience structural failure as the result of compression, tensile, bending or shearing forces [55,57,75], although generally it is strongest in compression and weakest in shear [116]. Bone deformation depends on the type of load applied, and micro-cracks will be orientated slightly differently depending on the type of bone tissue and the force applied [90,117]. It is therefore possible to identify the function of bone tools based on size and orientation of micro-cracks [38,101,111]. Compression and shearing fractures are usually the result of impact and seldom occur naturally [56] as mammalian long bones are adapted to compressive longitudinal loading [90,121]. The tensile strength and other mechanical properties of cortical bone may change with age and degree of mineralisation [122]. Bone implements used for different activities will undergo different mechanical stresses depending on the activity. For example, we would expect a bone projectile point to experience bending and shearing stresses on impact [123], whereas a bone wedge that has been hammered will undergo compressive stress. Bone points used as awls to perforate skins experience torsion and shearing forces [124]. It therefore follows that bone of a specific compositional nature will be better suited to a given task. The supposed arrowheads recovered from Howiesons Poort levels at Sibudu (B5d GS and C6d GS) therefore meet the morphological and mechanical requirements to perform this task; a hypothesis supported when coupled with the additional evidence of diagnostic impact fractures [36,38] and ‘retrieval’ cut marks.

Wang and colleagues [71] looked at the cortical bone fracture properties in five mammalian taxa, including humans, baboons (primates), canines (Carnivora), bovids (Artiodactyla) and rabbits (Lagomorpha), and found that fracture properties differed considerably between taxa due to variations in micro-structural and compositional properties. Canine bone had the highest fracture toughness values and bovid bone the lowest. These results conform to what is known about fracture properties of different cortical micro-structures. Unfortunately, apart from Wang’s [71] work very little research seems to have been done to ascertain bone mechanical properties of different animals, and so we have to rely on the equally few studies that have assessed mechanical properties of the different bone tissue types. This may, however, be over-simplistic in an actualistic setting as implements made from animal long bones might often incorporate two or more tissue structures, as is indeed evident at Sibudu.

The majority of specimens examined here contained high amounts of primary lamellar bone relative to plexiform fibro-lamellar and secondary bone. As has been mentioned above, primary lamellar bone is thought to be mechanically best adapted to impact stresses [13]. According to Ascenzi and colleagues’ [120] findings on primary bone elasticity, however, it is evident that the Sibudu worked bone shafts do not represent bone tissue of the greatest elastic properties, as longitudinal canals, which have the poorest elasticity, occur in the highest number of artefacts, whereas radial canals, which have the best elastic properties, occur in the lowest number. While elasticity is necessary for projectile weapons, it is not necessary for domestic utensils like awls. We do, however, see a greater number of bones exhibiting radial and reticular canals in the Howiesons Poort than we do in the post-Howiesons Poort. At a taxonomic level, we can see a mechanical distinction between cortical long bone from, say, Perissodactyla and Artiodactyla, with the former being more elastic. Faciley, these findings appear inconsistent with what we know about bone tool raw material selection in historic times, which favours Artiodactyla species for arrowheads [23,25]. However, based on limited microCT scans revealing bone arrowhead histography [101] it is evident that the percentage of Haversian tissue is greatly reduced or absent in the Holocene specimens. Based on these results it appears that people started experimenting in the post-Howiesons Poort period with bone from a different type of animal, perhaps from one of the many larger antelope abundant in the area, and that they had not yet learned to eliminate the weaker Haversian tissue during the manufacturing process. Another interesting feature of the post-Howiesons Poort sample at Sibudu is the complete absence of tools made from the bones of Perissodactyla. Because species of this taxon are well-represented in the unmodified fauna their absence in the modified category cannot be explained by environmental or demographic factors. Rather, it appears that there is a conscious avoidance of these animals in the younger assemblages.

Inferences about conscious decisions governing the choice of raw material selection are tenuous given the small sample size; yet, not wholly unwarranted. In many other parts of the world there is ample evidence demonstrating that cultural attitudes play a role in determining which animals are sourced for tools [29,124–126]. Deviations in selection patterning that does not arise from ecological or environmental changes can usually be ascribed to cultural factors [31,127]. Cultural choice may be inferred if selection preferentially favours a certain taxa, age, or skeletal element over others that are suitable for purpose [126]. While the choice of bone element is most often functionally driven, the choice of species is not [126]. Studies in North Africa have shown that during the Later Stone Age bone tools were made from only a small section of the animal taxa present at the site, suggesting that people embedded bone tools within culturally-mediated technological strategies [128]. Similarly, in the South African Iron Age, certain taxa seem to have been deliberately avoided, although mechanical suitability in addition to cultural choice may have been factor [129]. Although the results of the present study hint at preferential selection, it is uncertain whether the apparent shift away from Perissodactyla to Artiodactyla at Sibudu is representative of the entire worked bone sample recovered from the site.

Whether the results presented here can tell us anything about human agency governing bone tool raw material selection strategies at Sibudu is hampered by two factors: 1) the small sample size that was available and suitable for micro-CT analysis, and 2) the limited published studies characterising bone histology of southern African mammals. It is unfortunate that many of the tools presented here could not be confidently identified to taxa. Collagen isotope analysis may hold potential for the future. Nevertheless, I have shown that the people living at Sibudu between ~65 to ~58 ka were fashioning tools out of animal bones brought in as food items, and were not necessarily hunting ‘exotic’ animals for raw material. The apparent shift in taxon focus from perissodactyls in the Howiesons Poort to artiodactyls in the post-Howiesons Poort and beyond is concomitant with other changes evinced during this period of environmental transition [6,34].

## Supporting information

S1 File Powerpoint presentation showing transverse micro-CT scans for each piece of bone mentioned in the article. (PPTX)
