# Supplementary material for: Identifying animal taxa used to manufacture bone tools during the Middle Stone Age at Sibudu, South Africa: Results of a CT-rendered histological analysis
Source: PLoS One. 2018 Nov 29;13(11):e0208319. doi: 10.1371/journal.pone.0208319 (PMC6264865; doi:10.1371/journal.pone.0208319)

## Slide 1
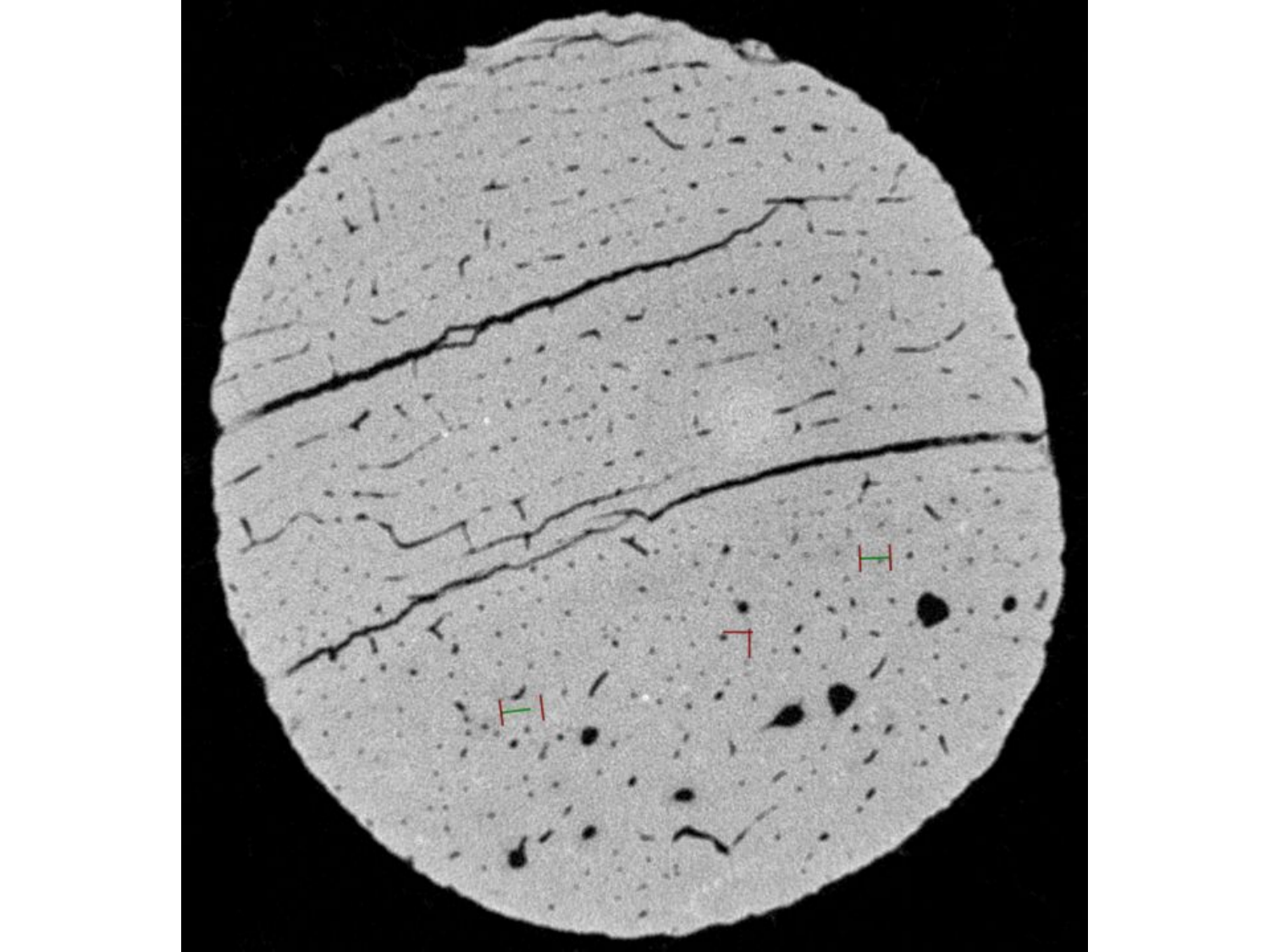

## Slide 2
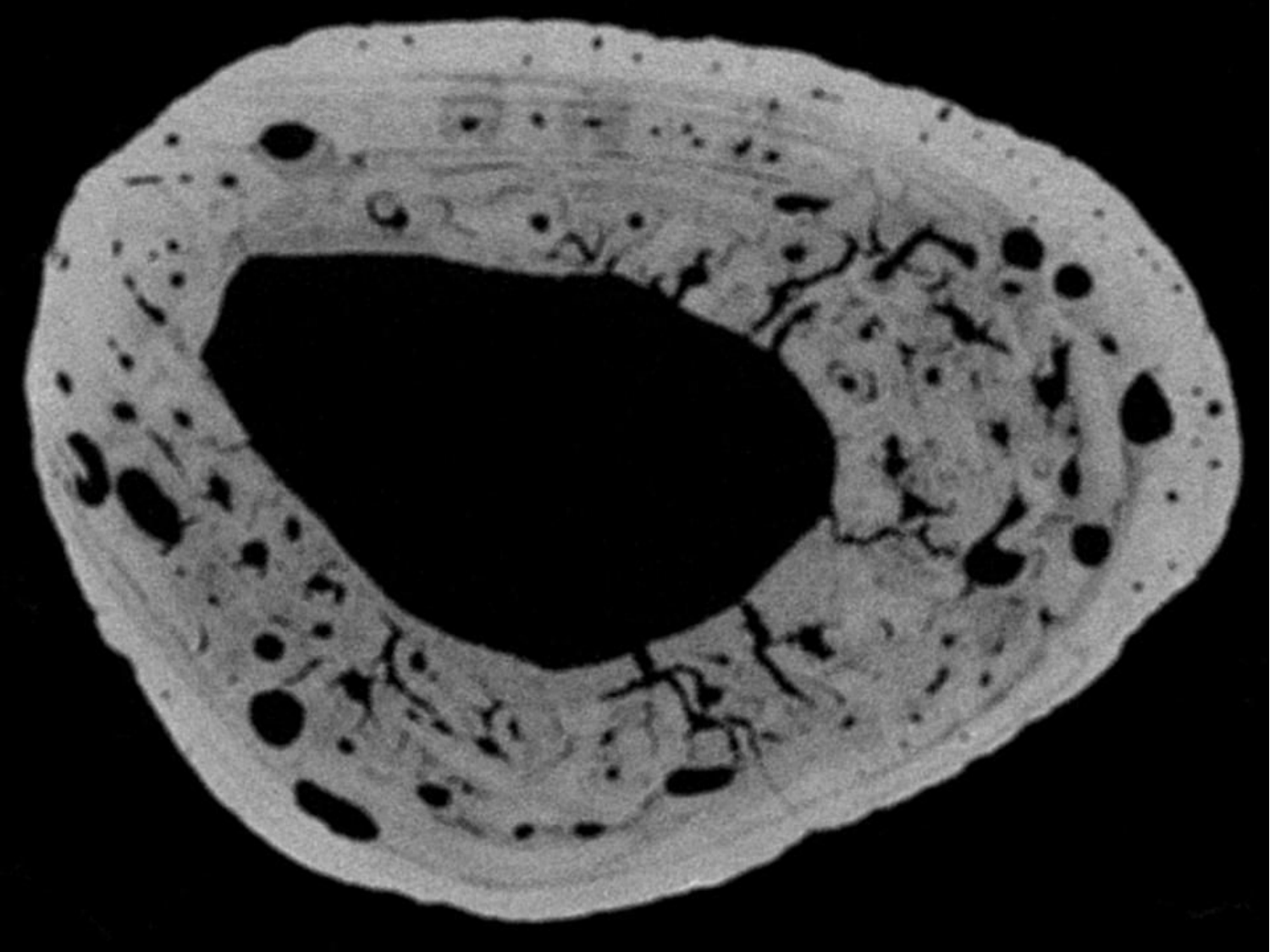

## Slide 3
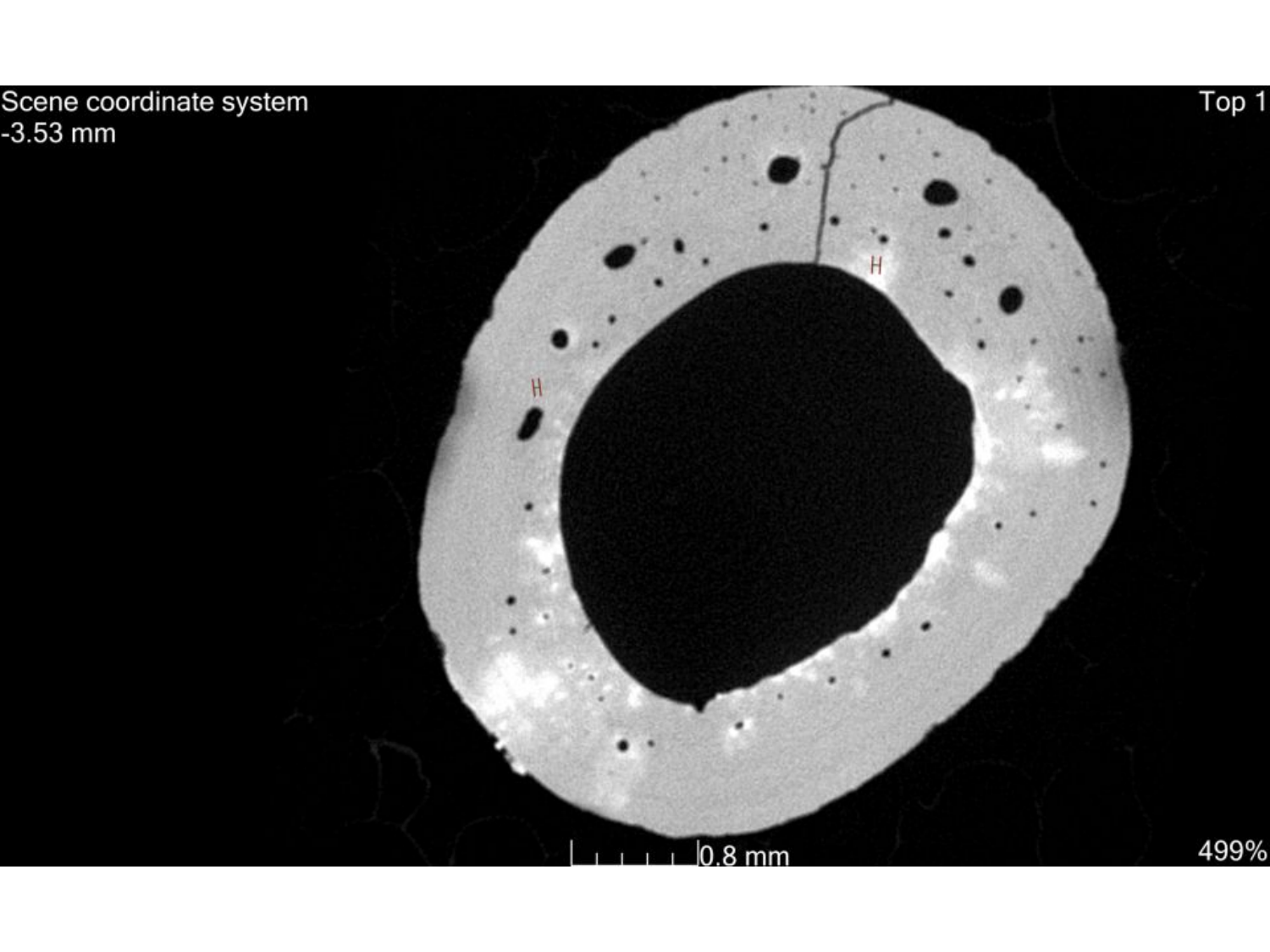

## Slide 4
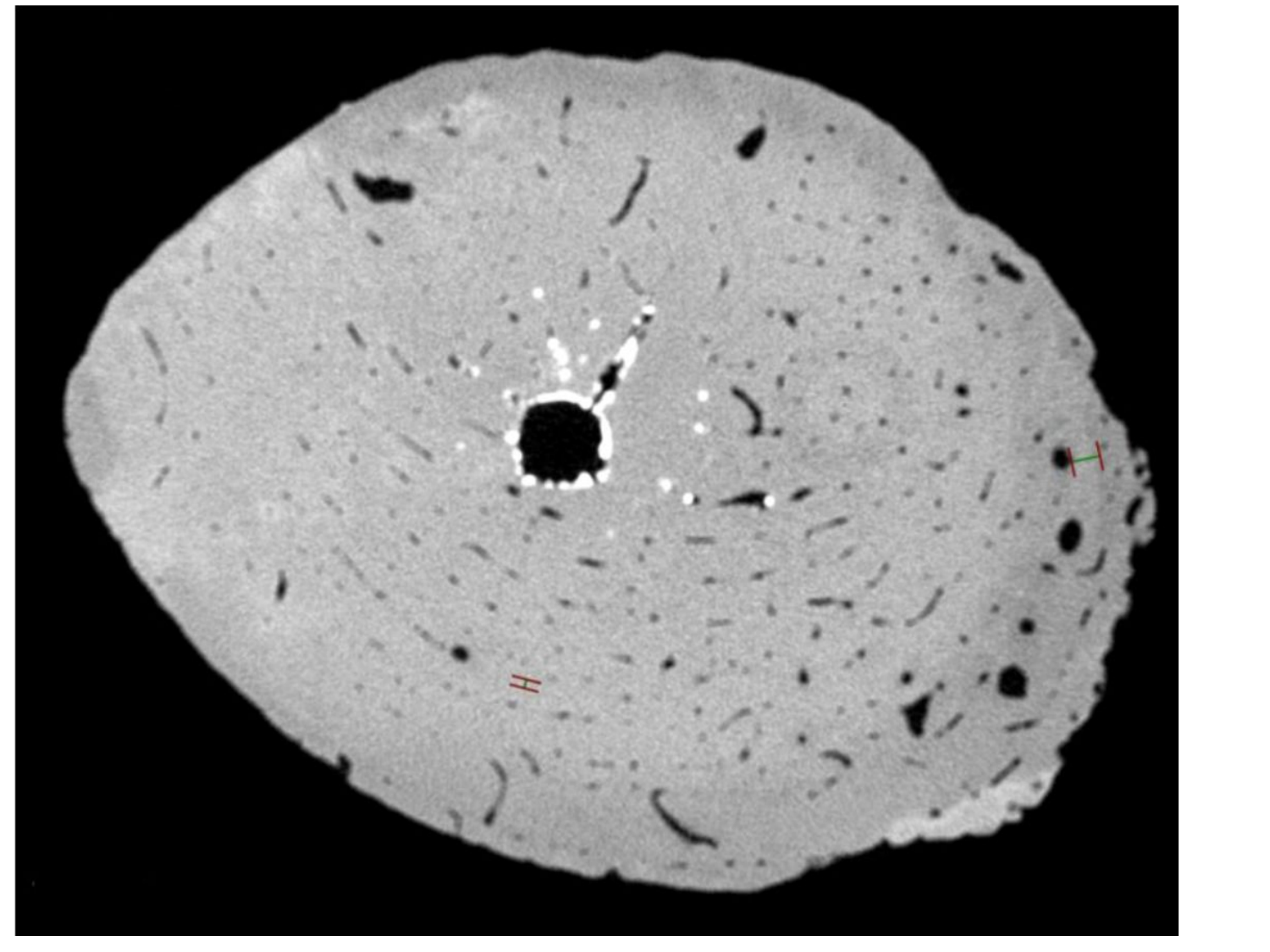

## Slide 5
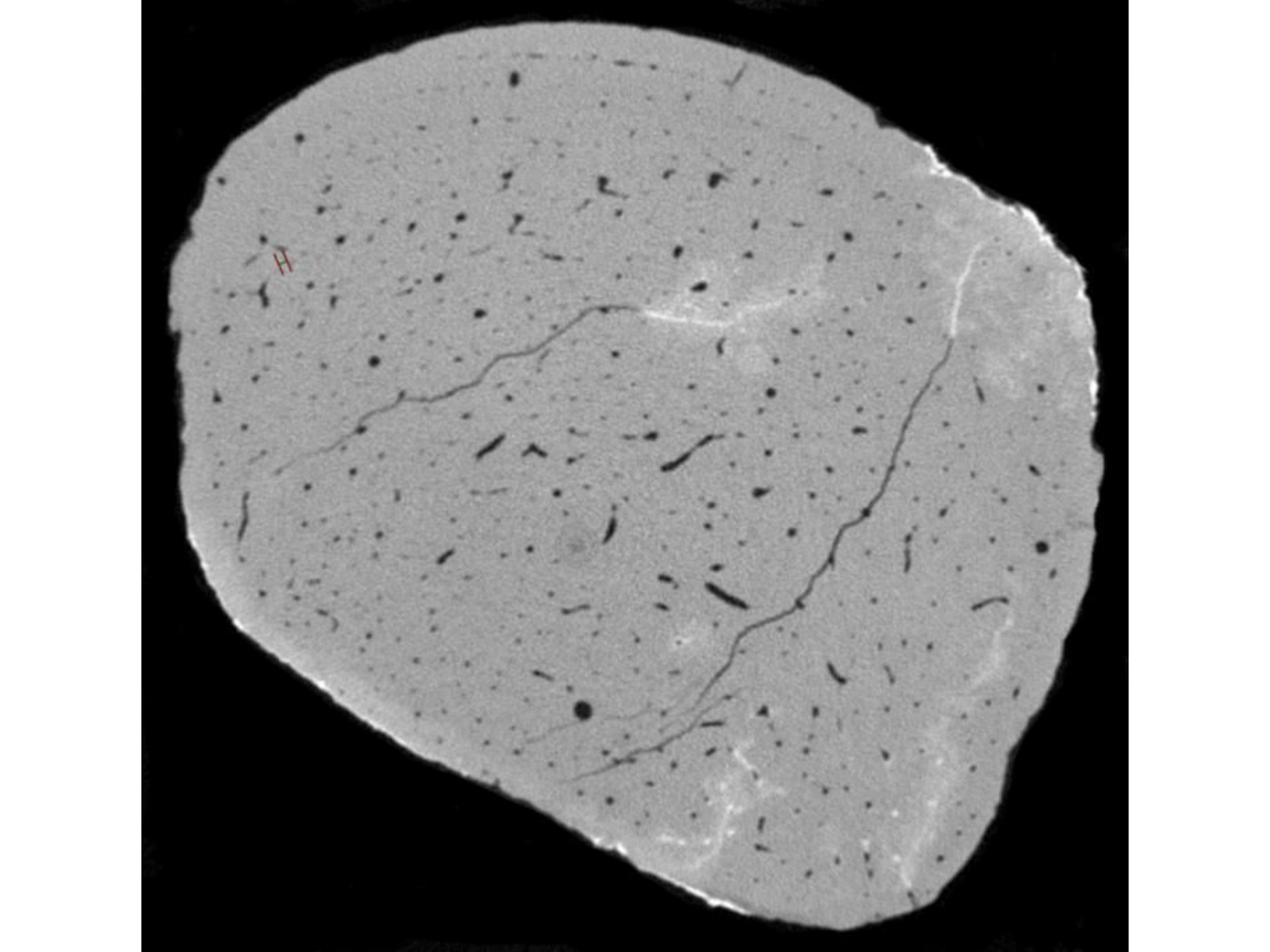

## Slide 6
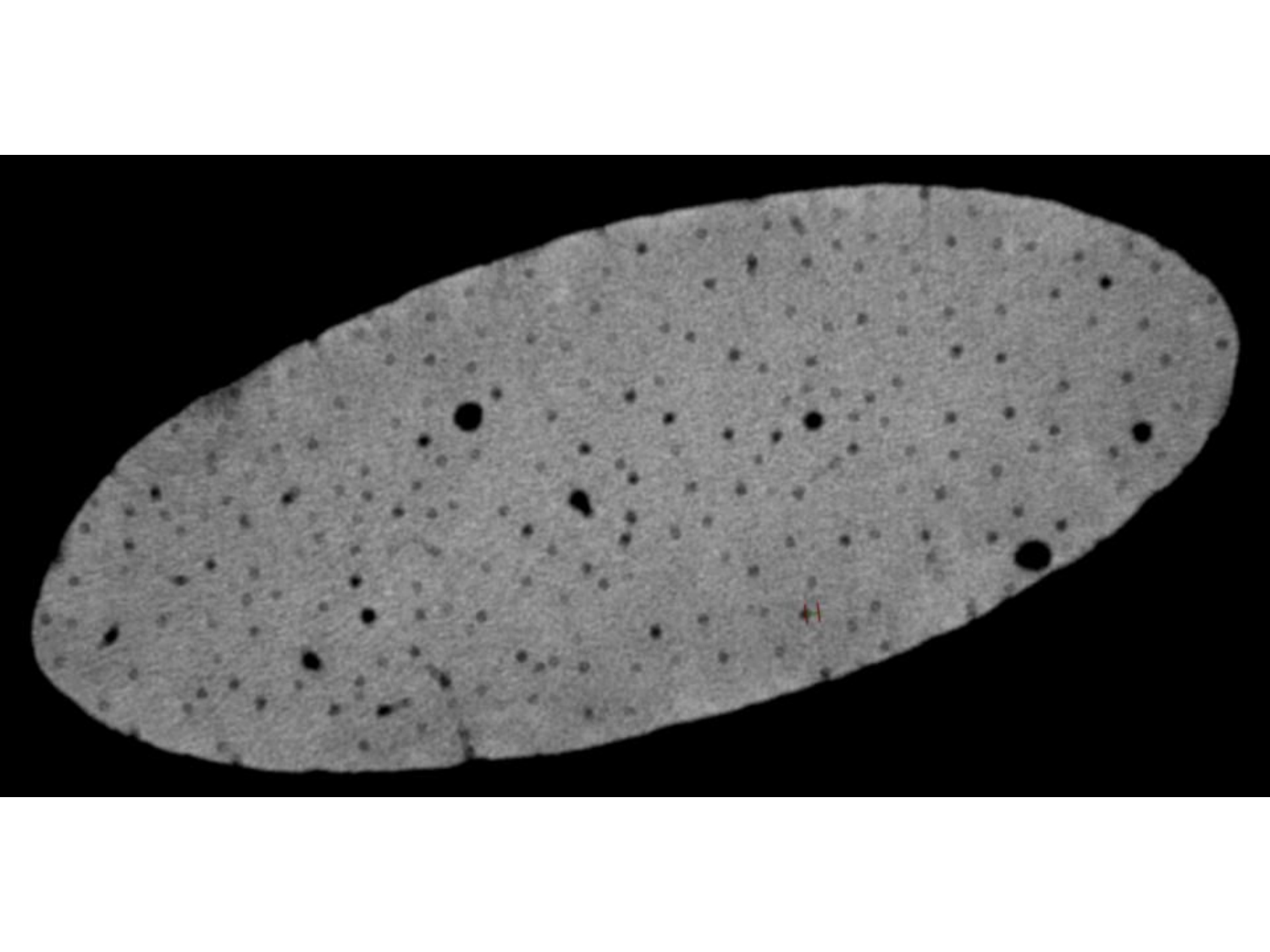

## Slide 7
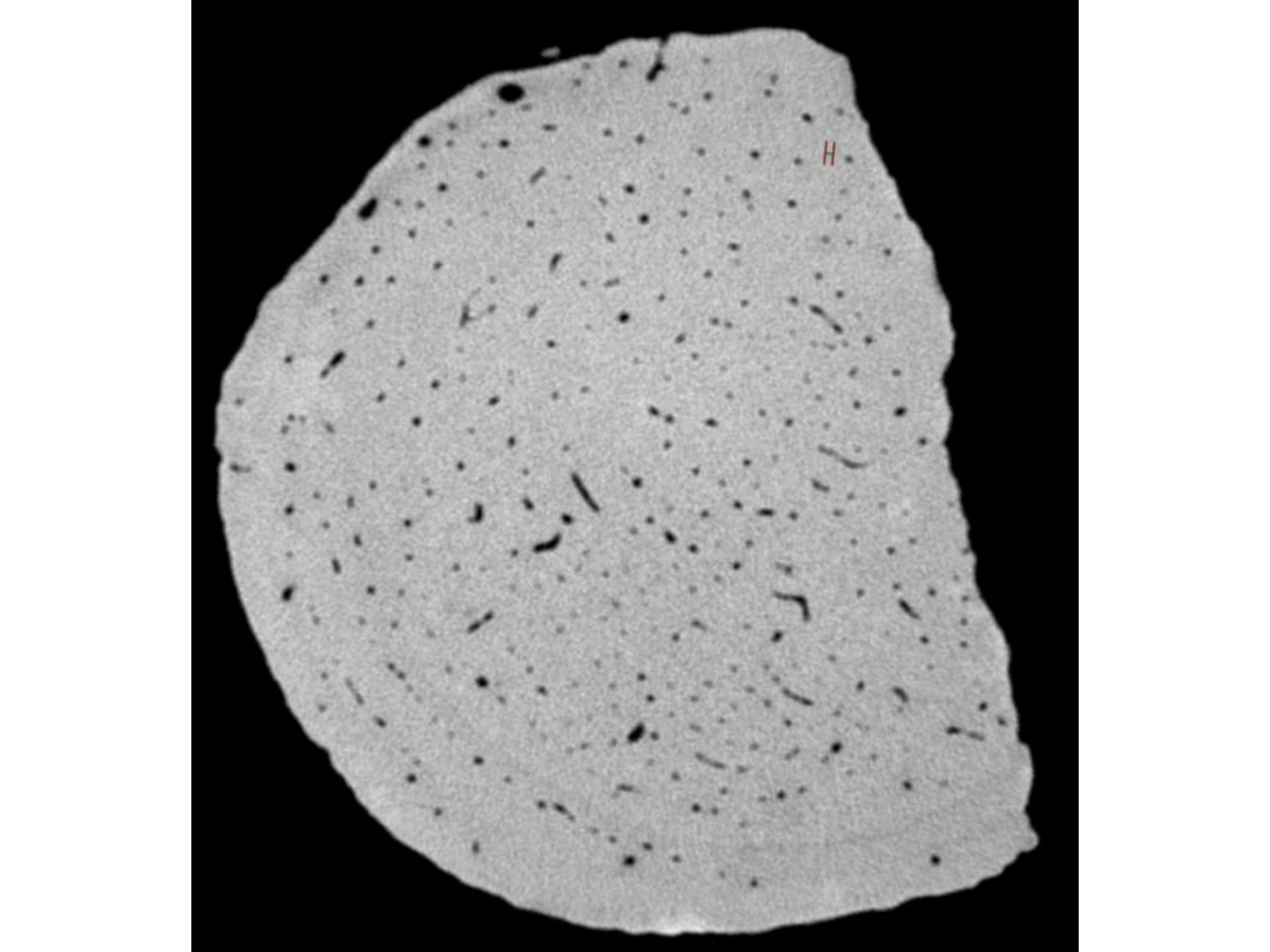

## Slide 8
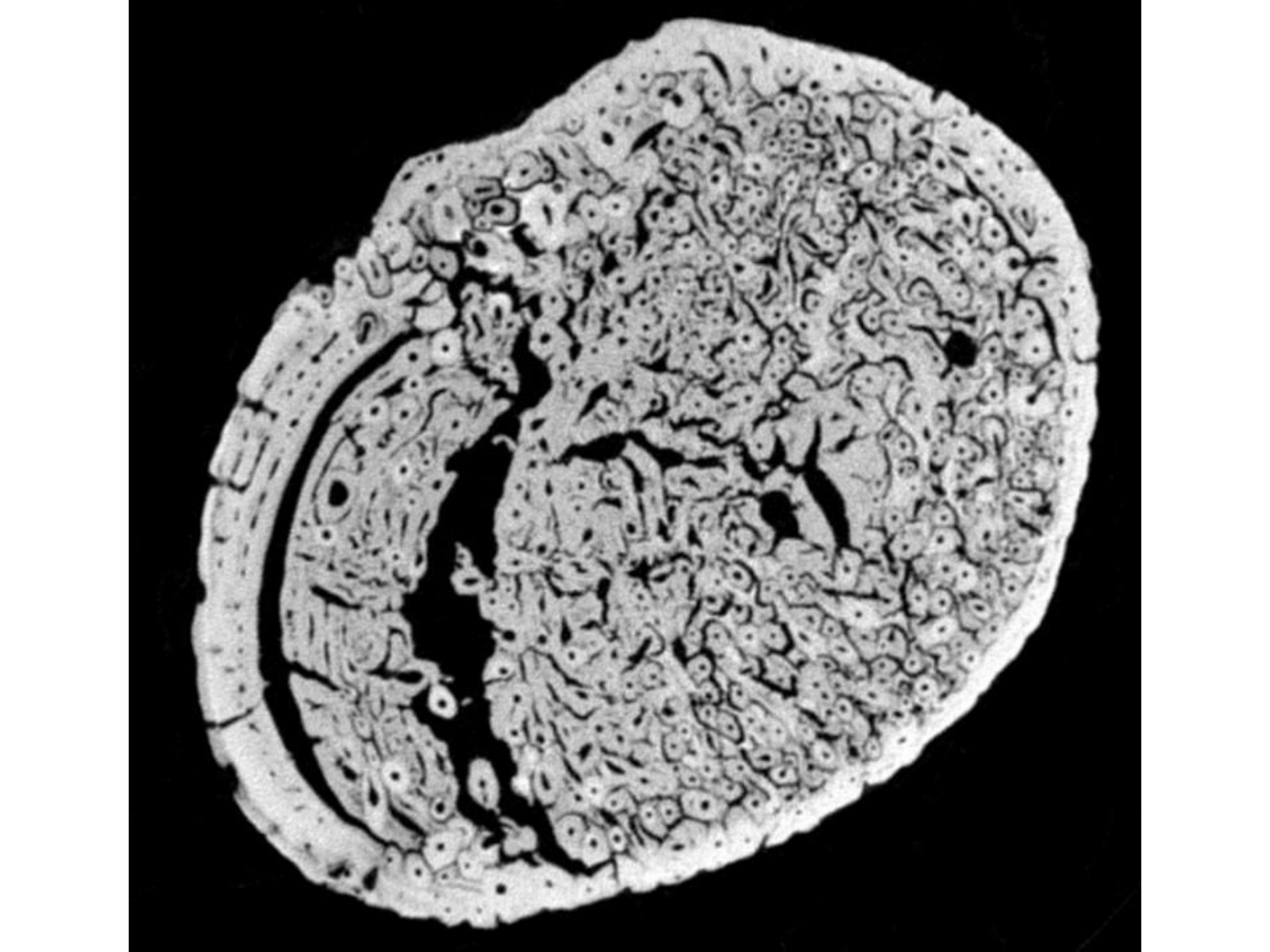

## Slide 9
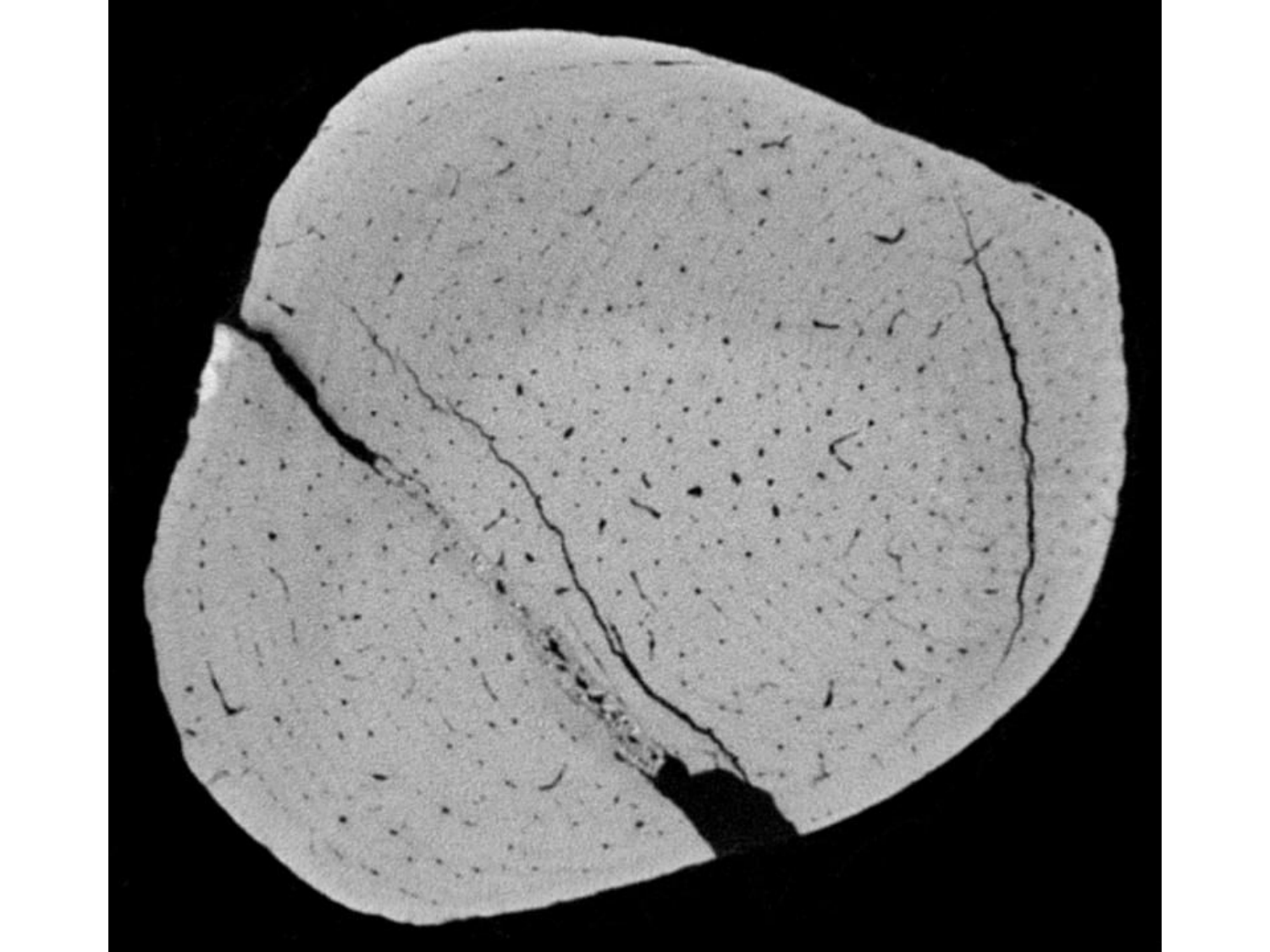

## Slide 10
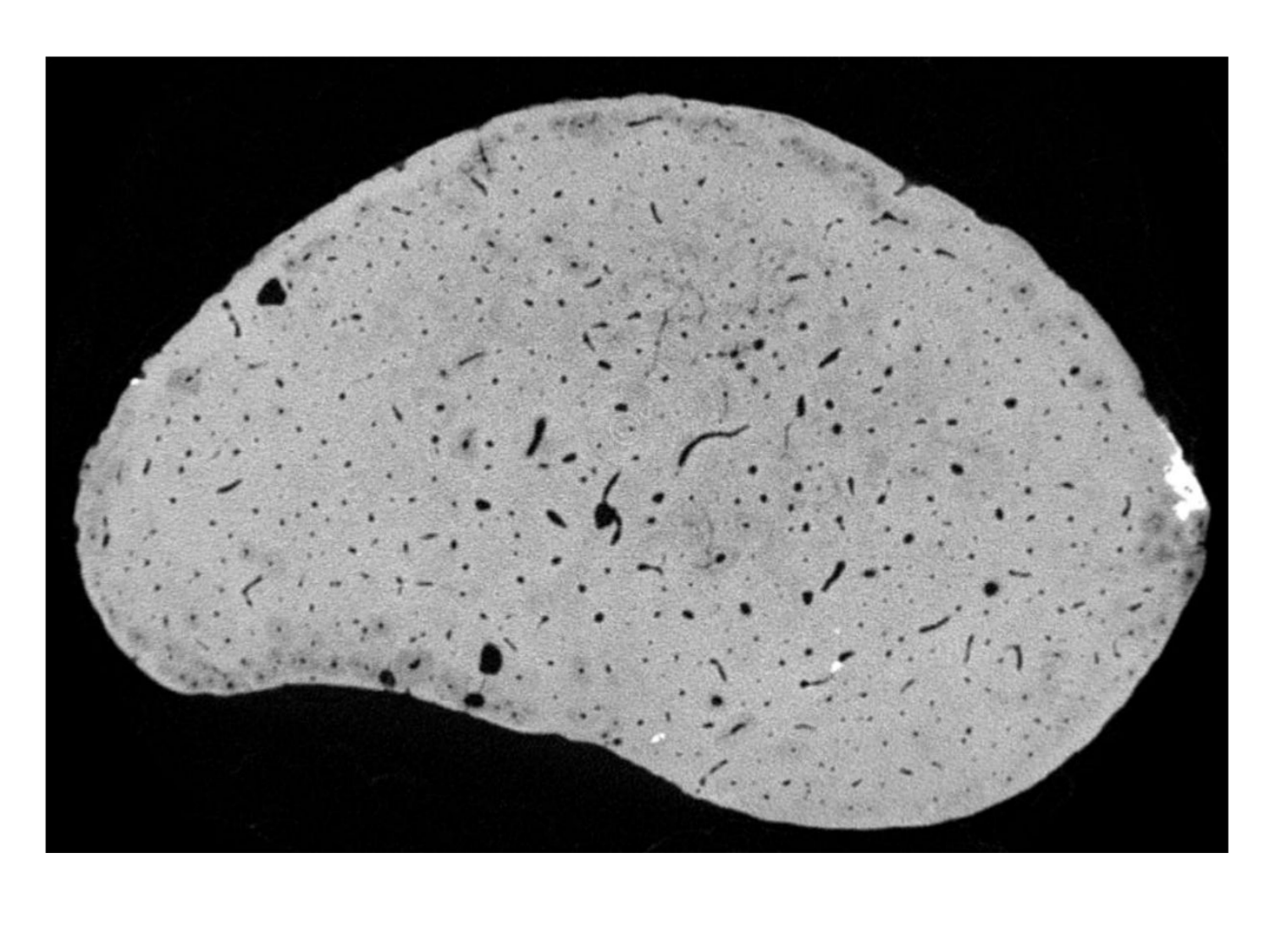

## Slide 11
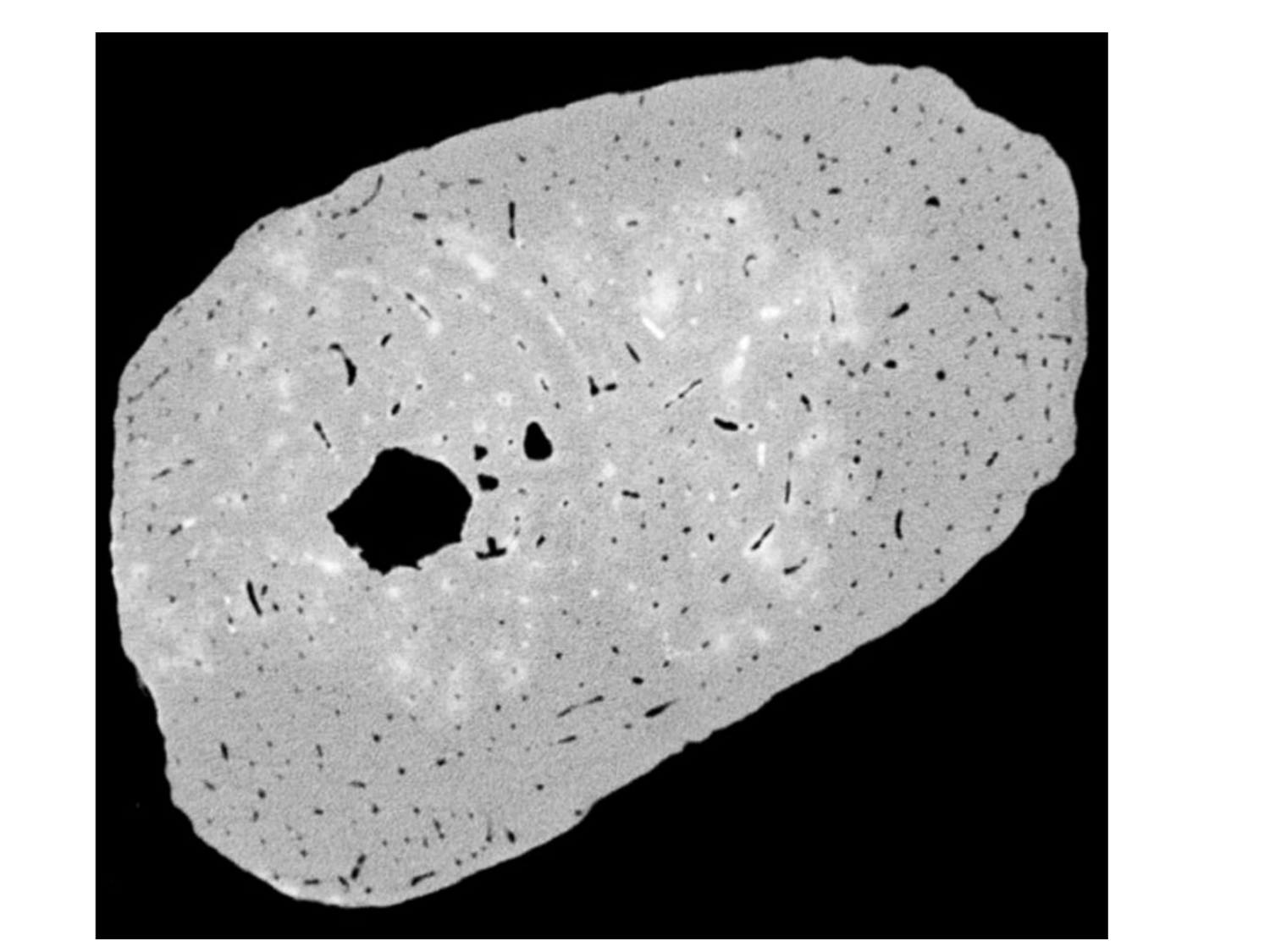

## Slide 12
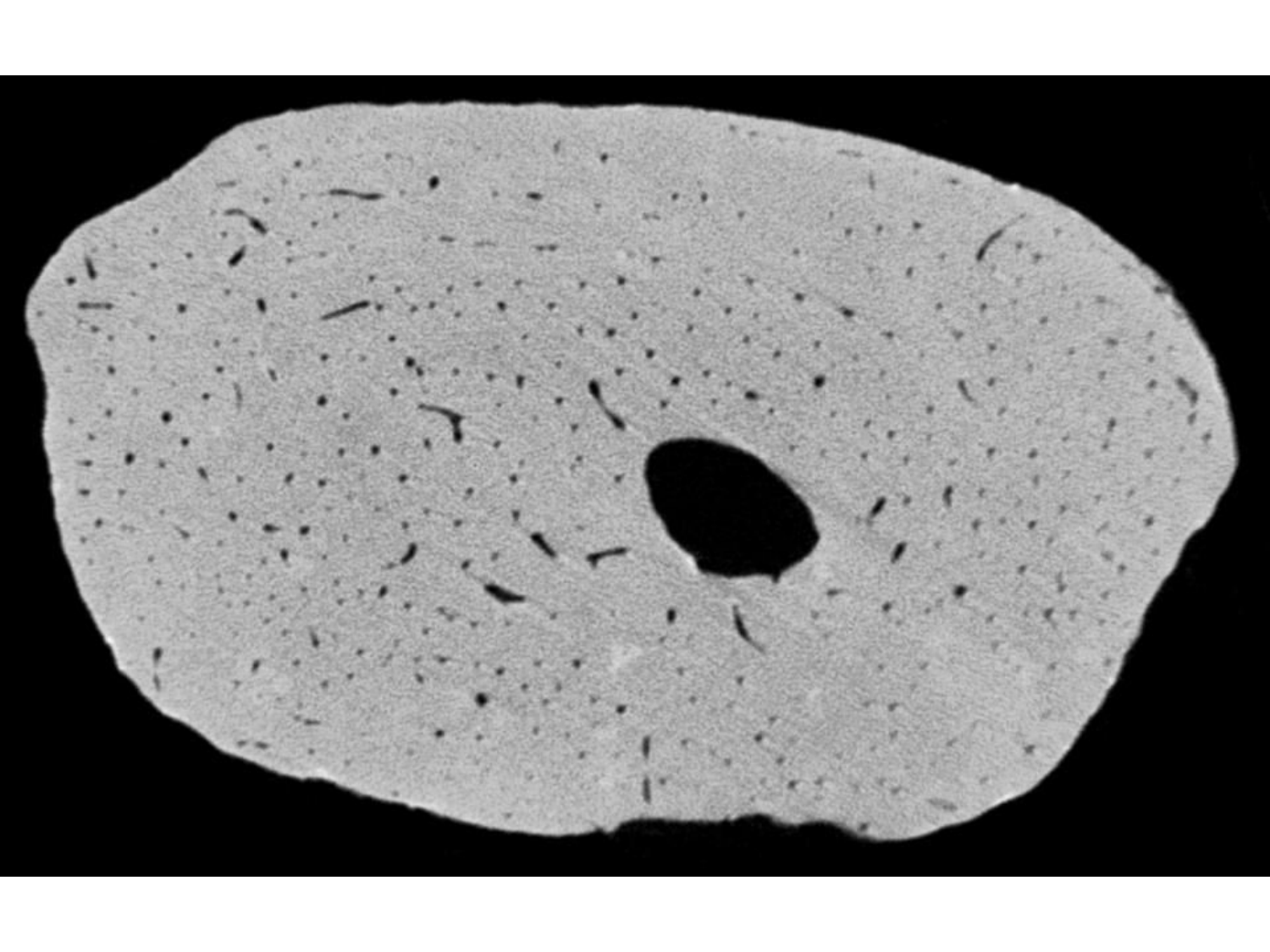

## Slide 13
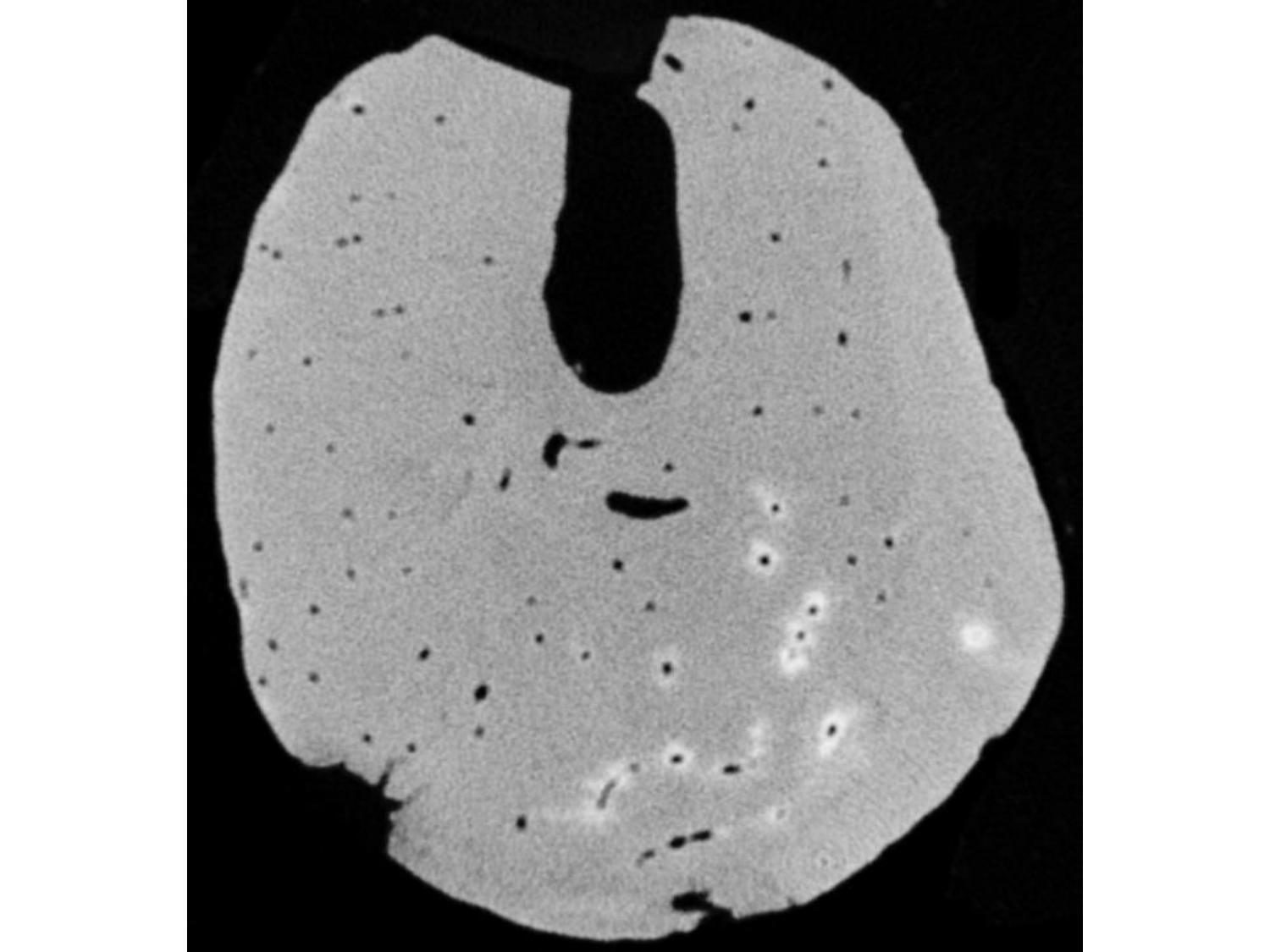

## Slide 14
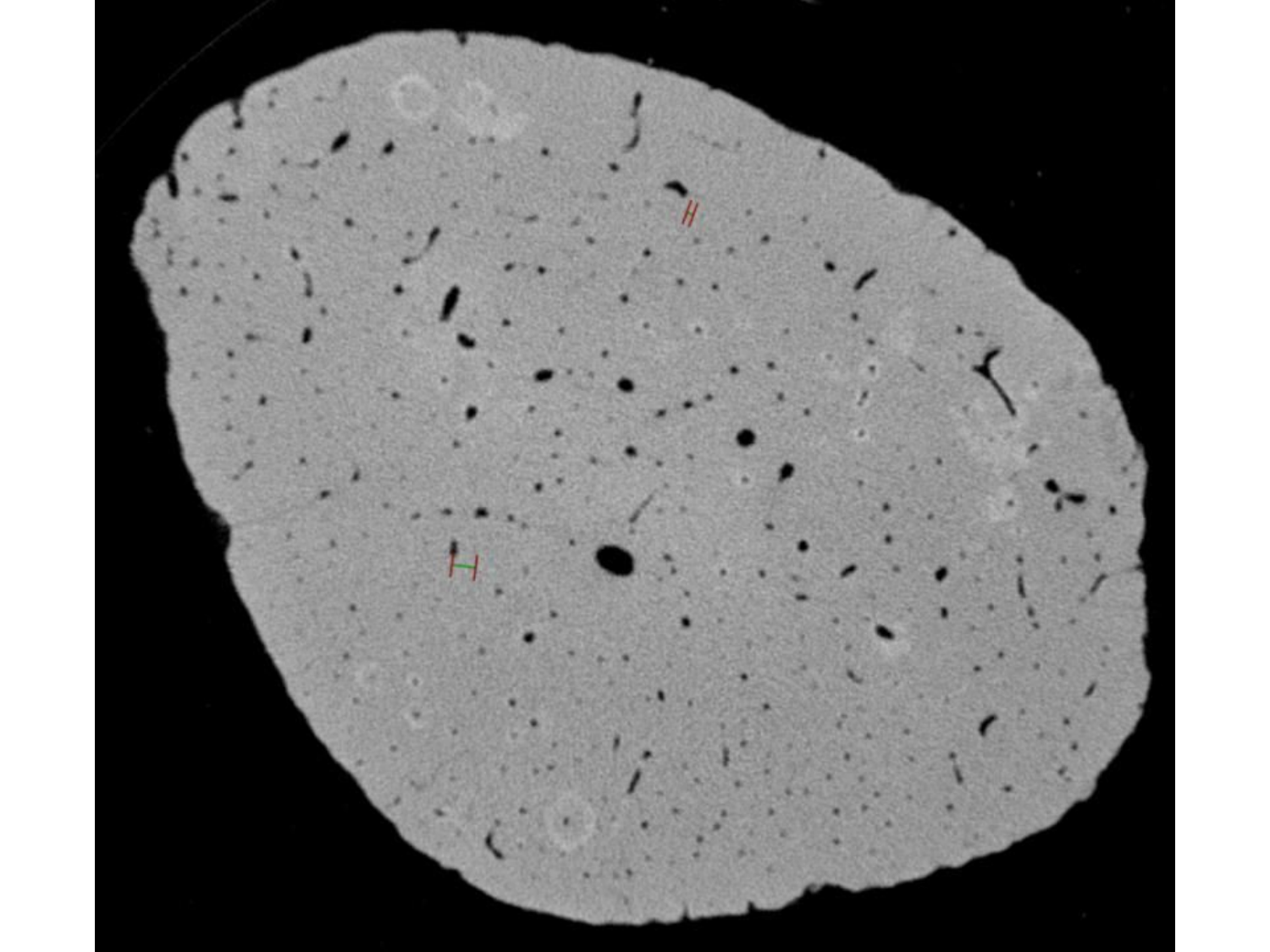

## Slide 15
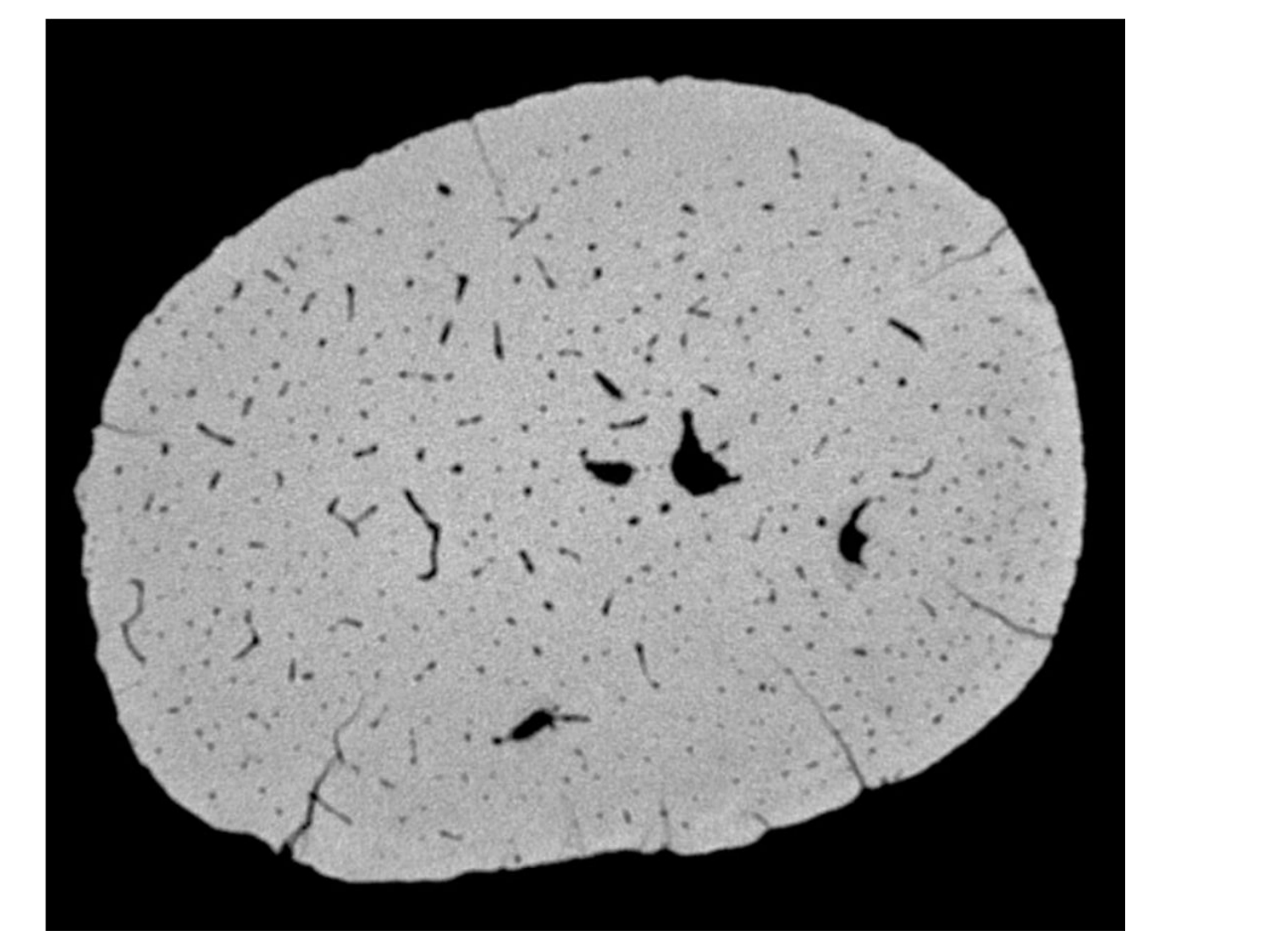

## Slide 16
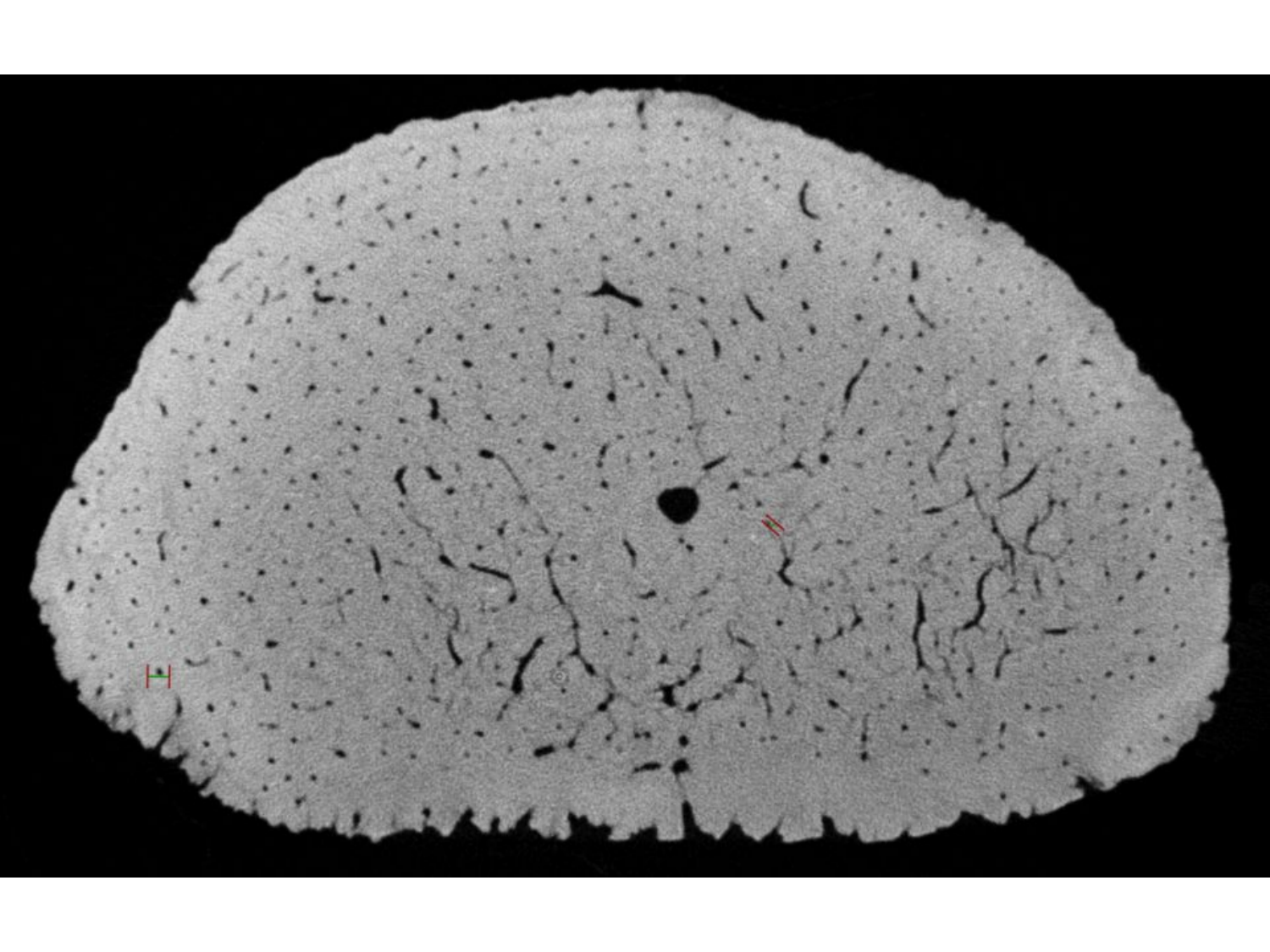

## Slide 17
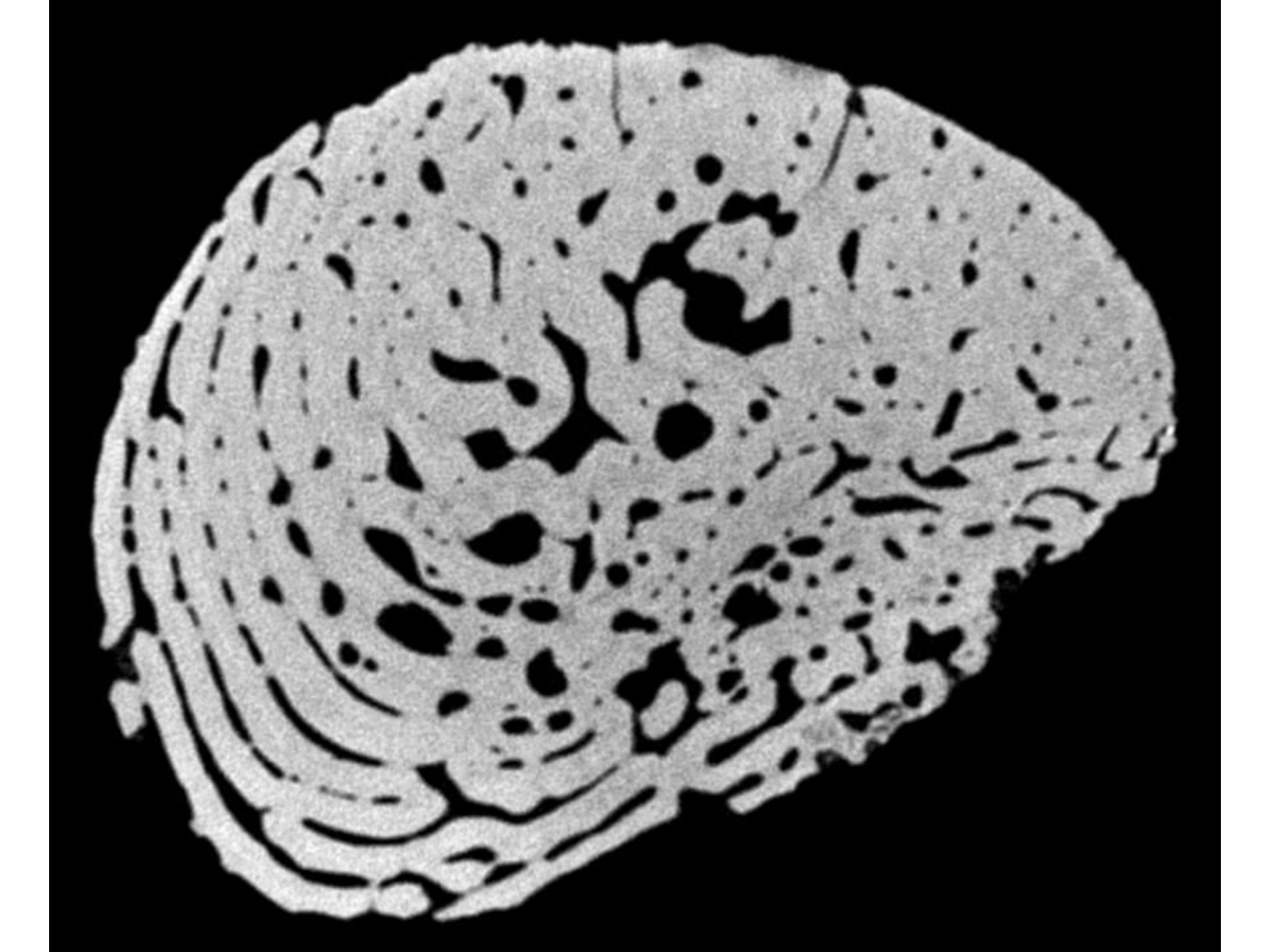

## Slide 18
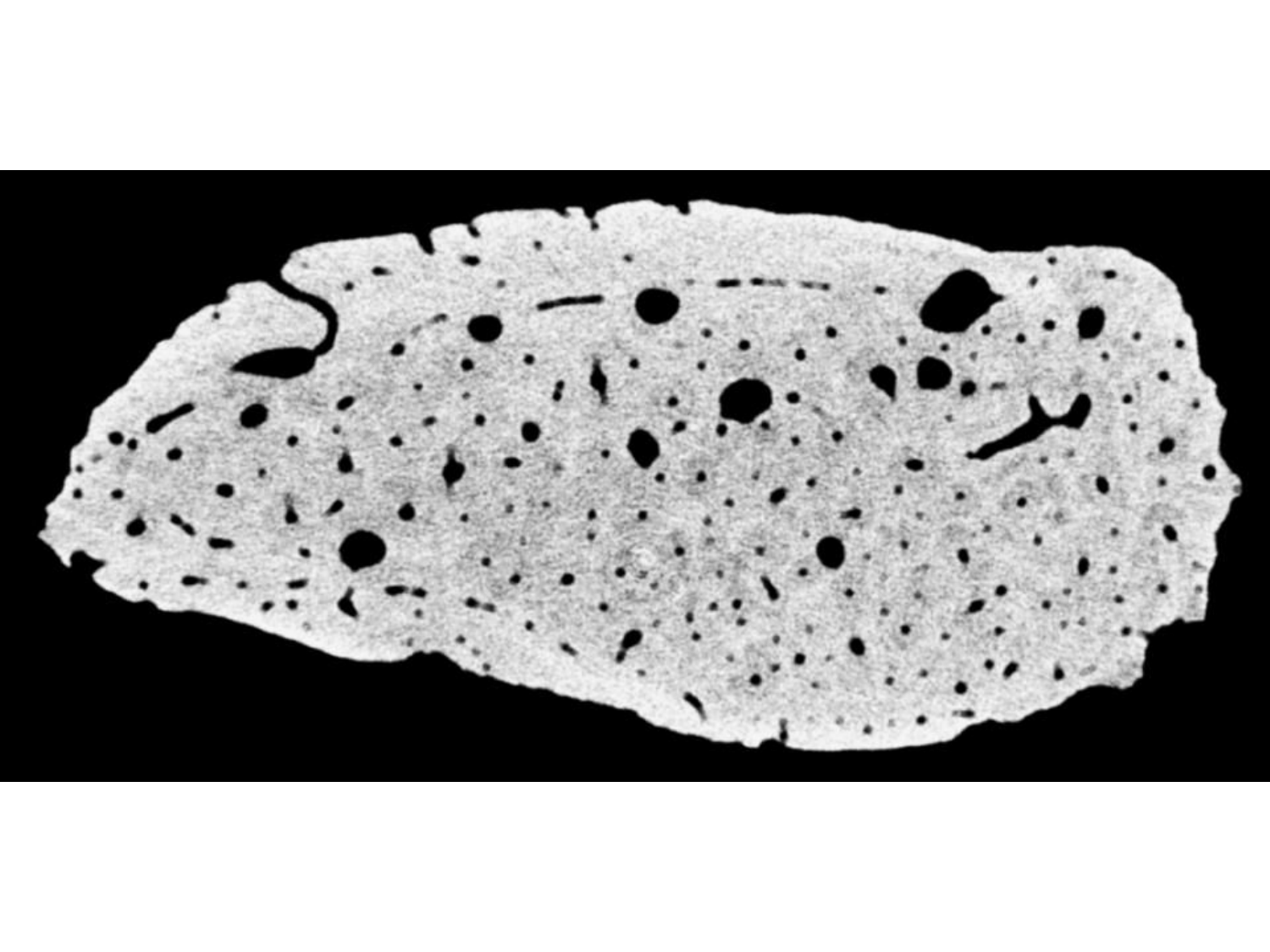

## Slide 19
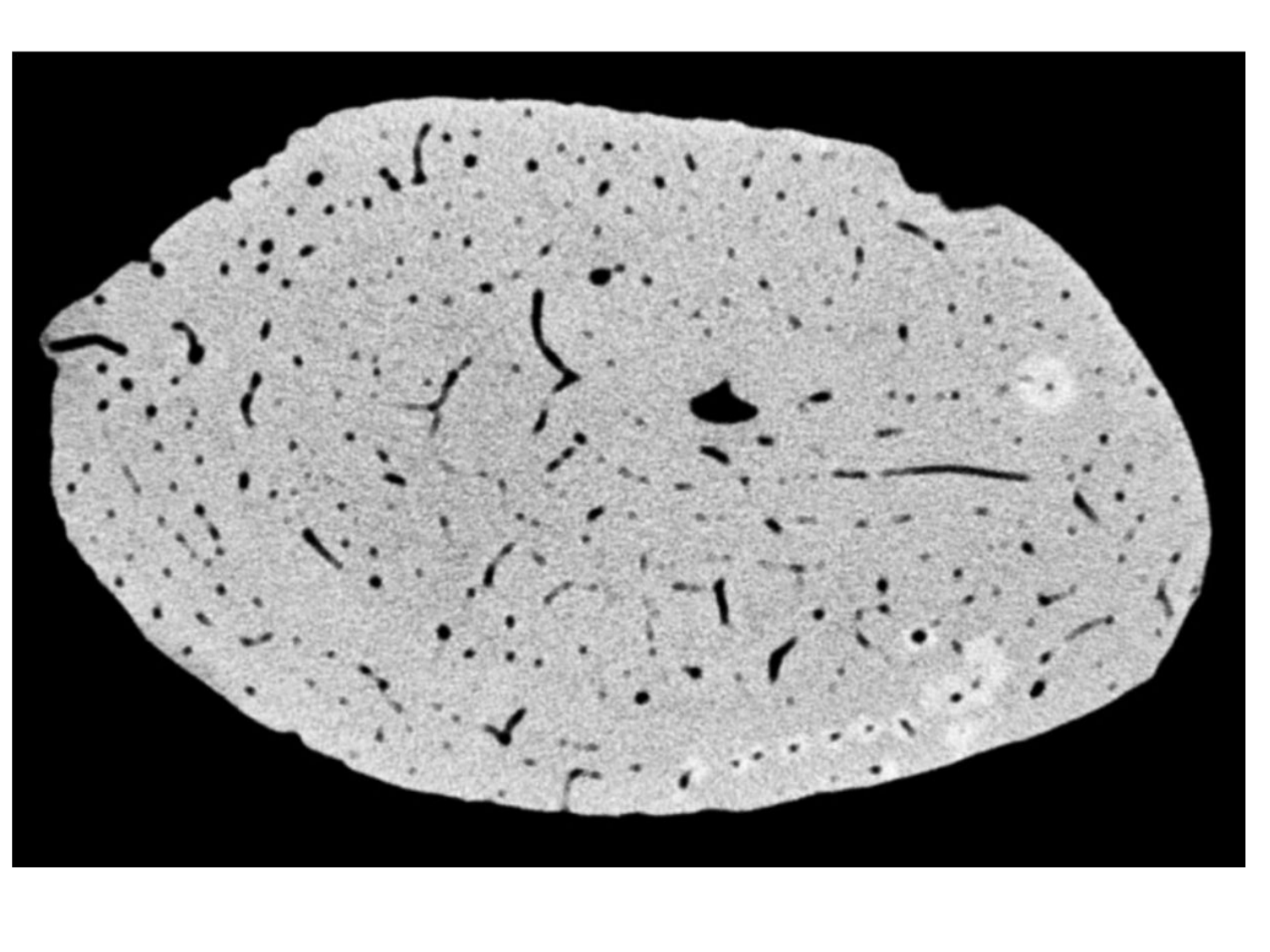

## Slide 20
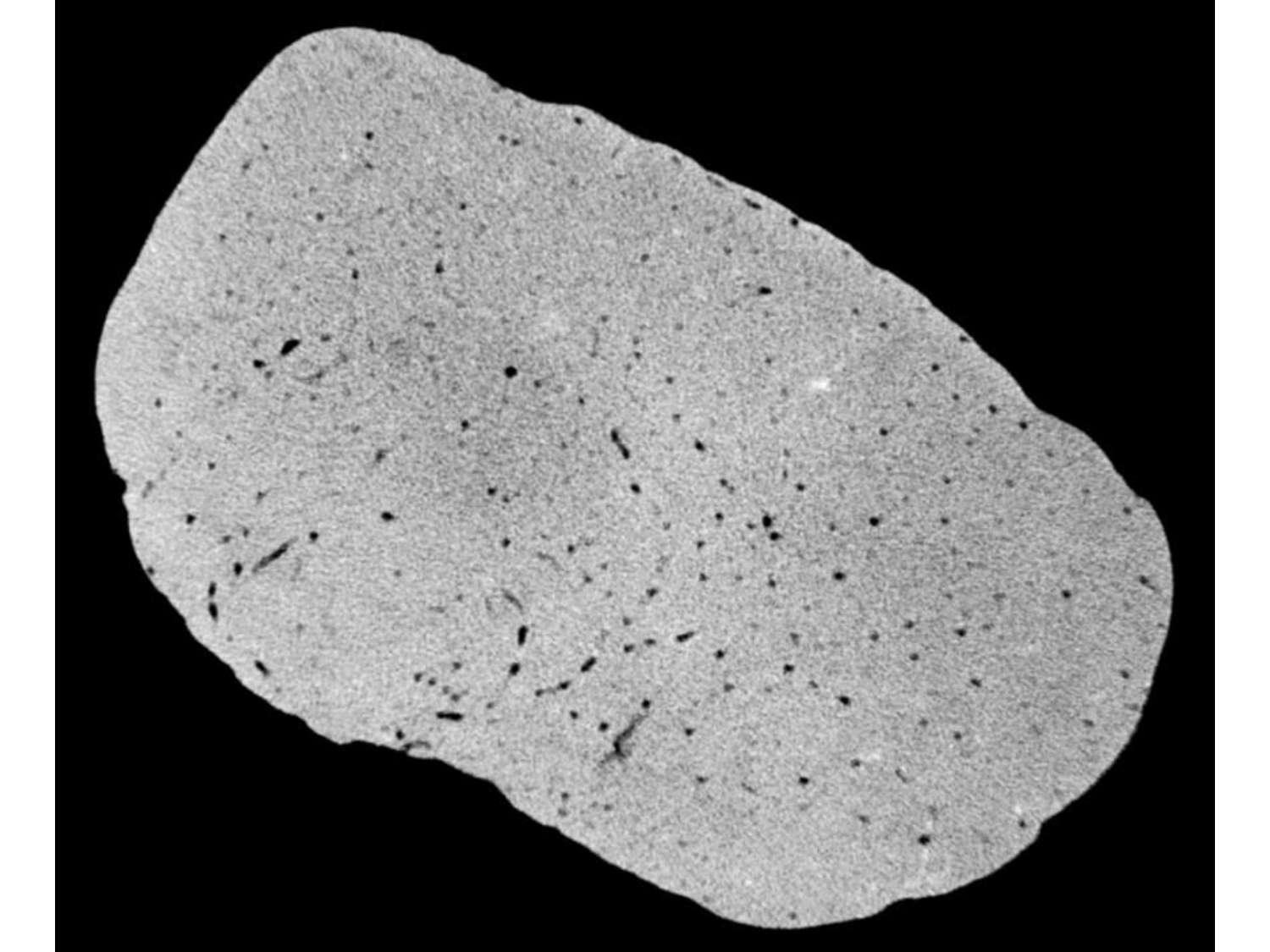

## Slide 21
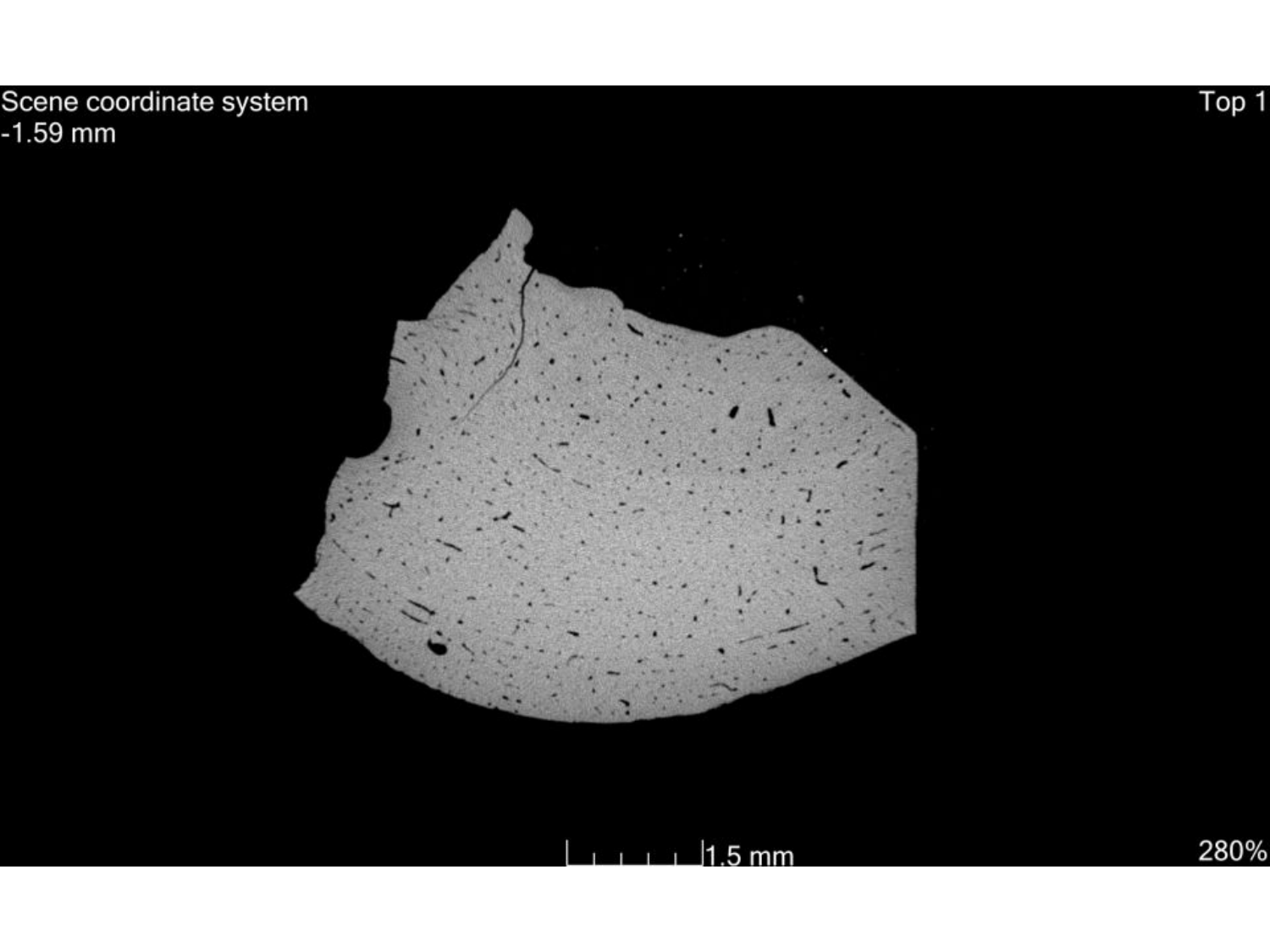

## Slide 22
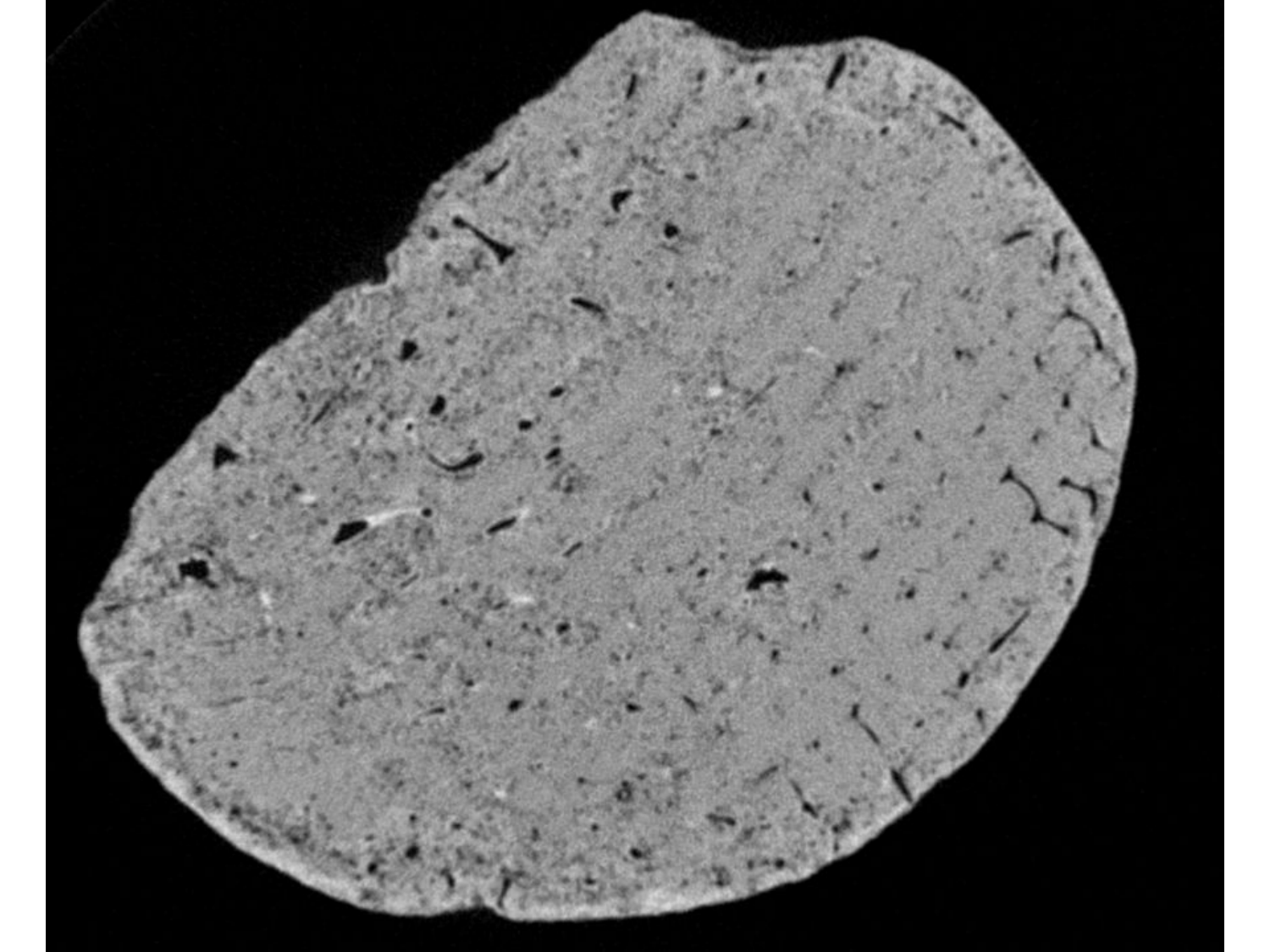

## Slide 23
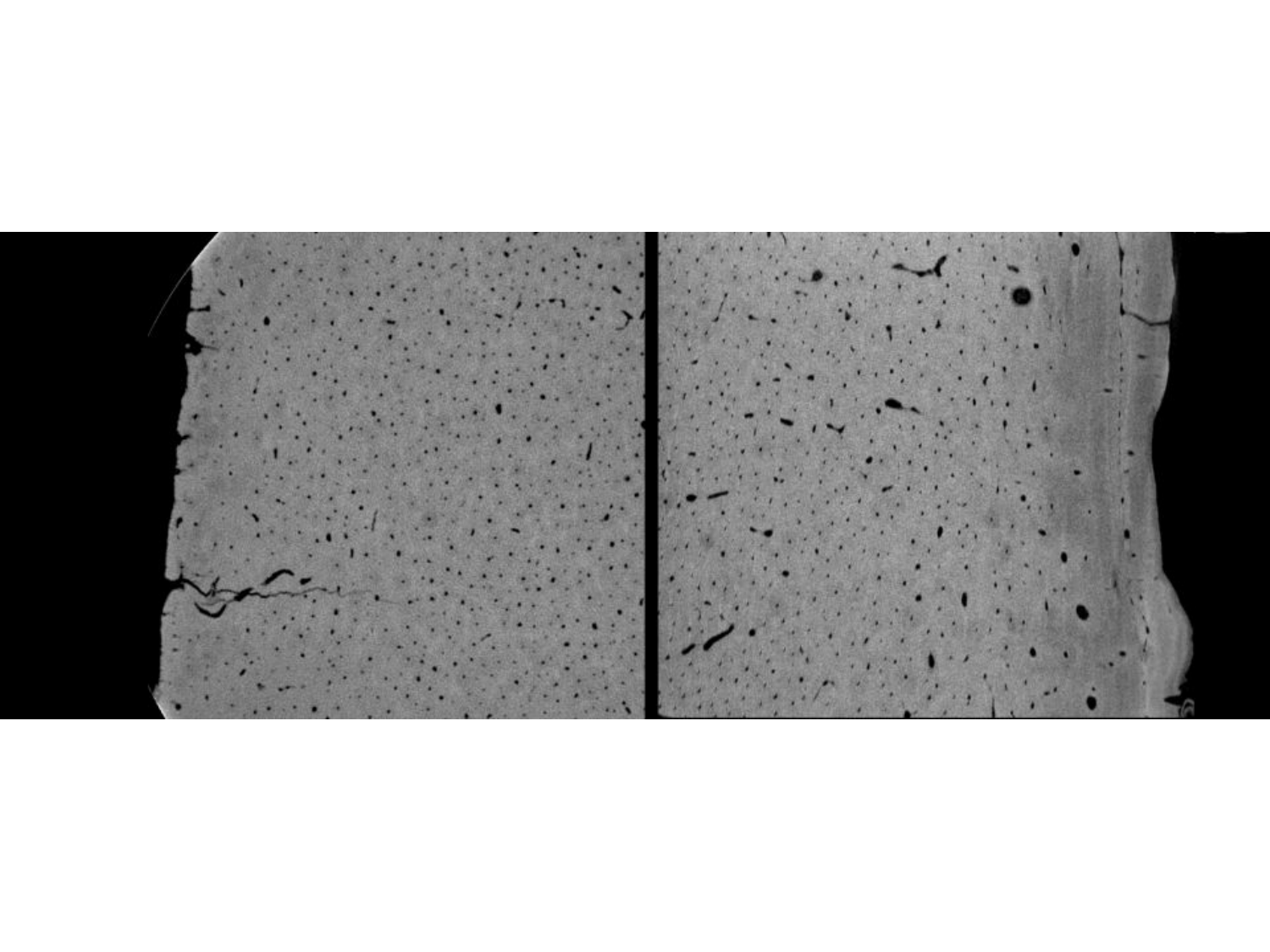

## Slide 24
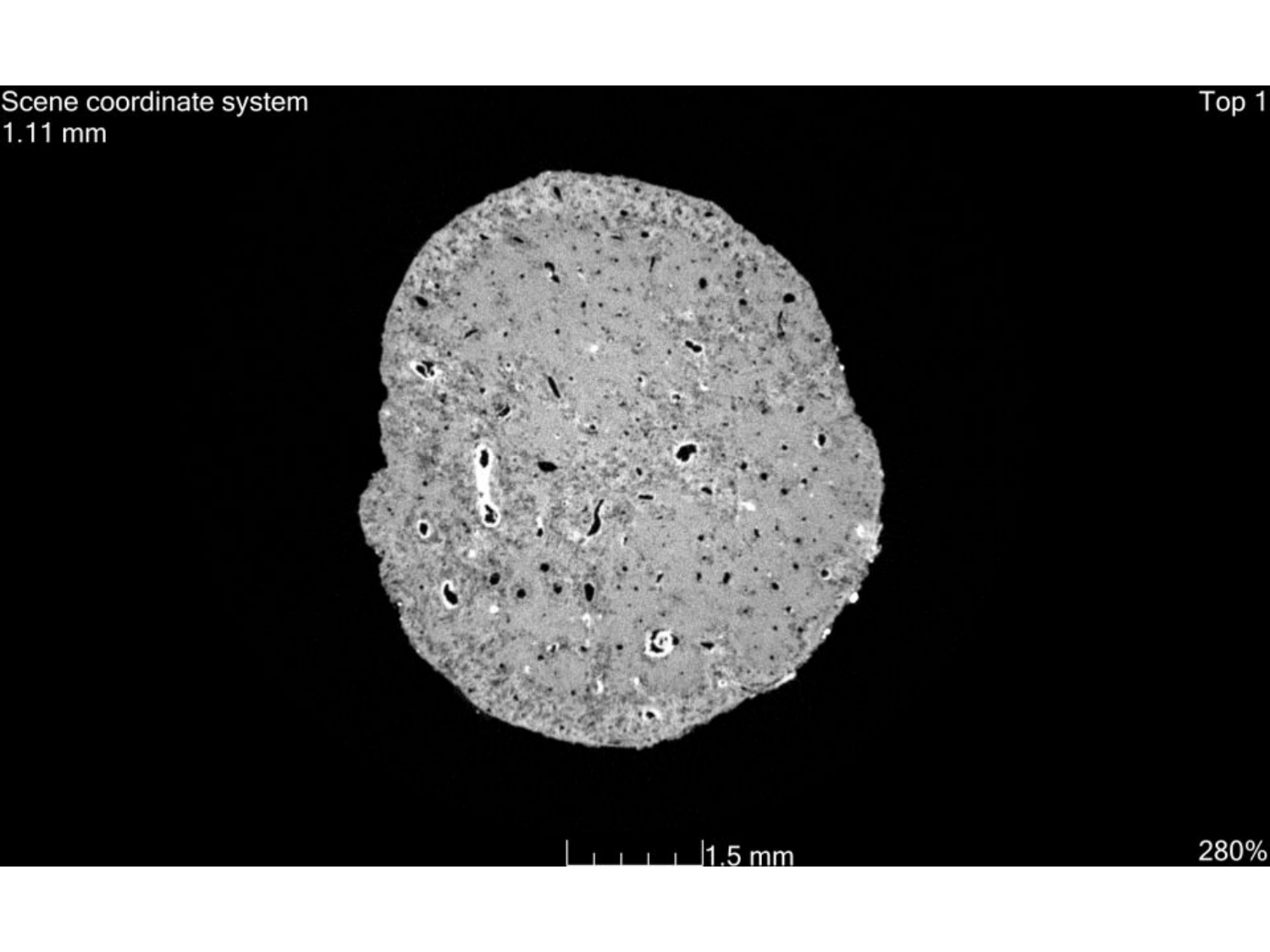

Supplement: S1 File — (PPTX) [file pone.0208319.s001.pptx]
